# Secondary Metabolites of the Genus *Amycolatopsis*: Structures, Bioactivities and Biosynthesis

**DOI:** 10.3390/molecules26071884

**Published:** 2021-03-26

**Authors:** Zhiqiang Song, Tangchang Xu, Junfei Wang, Yage Hou, Chuansheng Liu, Sisi Liu, Shaohua Wu

**Affiliations:** Yunnan Institute of Microbiology, School of Life Sciences, Yunnan University, Kunming 650091, China; songzhiqiang@mail.ynu.edu.cn (Z.S.); xu2950129@163.com (T.X.); wang_junfei@163.com (J.W.); houyage@126.com (Y.H.); liucs313@126.com (C.L.); liusisi1994@126.com (S.L.)

**Keywords:** **Actinomycetes**, *Amycolatopsis*, antibiotics, natural products, chemical structures, biological activities, biosynthetic pathways

## Abstract

*Actinomycetes* are regarded as important sources for the generation of various bioactive secondary metabolites with rich chemical and bioactive diversities. *Amycolatopsis* falls under the rare actinomycete genus with the potential to produce antibiotics. In this review, all literatures were searched in the Web of Science, Google Scholar and PubMed up to March 2021. The keywords used in the search strategy were “*Amycolatopsis*”, “secondary metabolite”, “new or novel compound”, “bioactivity”, “biosynthetic pathway” and “derivatives”. The objective in this review is to summarize the chemical structures and biological activities of secondary metabolites from the genus *Amycolatopsis*. A total of 159 compounds derived from 8 known and 18 unidentified species are summarized in this paper. These secondary metabolites are mainly categorized into polyphenols, linear polyketides, macrolides, macrolactams, thiazolyl peptides, cyclic peptides, glycopeptides, amide and amino derivatives, glycoside derivatives, enediyne derivatives and sesquiterpenes. Meanwhile, they mainly showed unique antimicrobial, anti-cancer, antioxidant, anti-hyperglycemic, and enzyme inhibition activities. In addition, the biosynthetic pathways of several potent bioactive compounds and derivatives are included and the prospect of the chemical substances obtained from *Amycolatopsis* is also discussed to provide ideas for their implementation in the field of therapeutics and drug discovery.

## 1. Introduction

Antibiotics produced by microorganisms have made a significant contribution to human health. Among them, *Actinomycetes* are the most important sources for drug lead compounds. However, researchers have been turned to rare *Actinomycetes* to develop novel antibiotics with the emergence of multidrug-resistant bacteria [1]. In 1986, Lechevalier et al. defined *Amycolatopsis* as a new genus to accommodate nocardioform *Actinomycetes* having type IV cell wall composition and lacking mycolic acids [2]. Up to now, by searching in the List of Prokaryotic names with Standing in Nomenclature website (http://www.bacterio.net, accessed on 20 September 2020), this genus covered 94 verified species and 4 subspecies, and forms a unique branch in the evolutionary tree of Pseudonocardiaceae. Among 26 species covered in this review, most of them colonize in a wide variety of soil and a few species survival in terrestrial (insect, lichen, island, plant) and marine (sponge, sediment) environment. The various habitats allow *Amycolatopsis* to produce abundant secondary metabolites.

The genus *Amycolatopsis* is regarded as an important source of diverse valuable bioactive natural products covering many antibiotics [3]. The most notable antibiotics produced by *Amycolatopsis* strains include rifamycin [4] and vancomycin [5]. In the early 1950s, vancomycin had been first extracted from *Amycolatopsis orientalis* that was originally regarded as *Streptomyces orientalis* [6]. Vancomycin was introduced for clinical use in 1958 and sparsely used during the first 30 years of its introduction, due to its fewer advantages over semisynthetic antibiotics like penicillin, cephalosporin, lincomycin, and fluoroquinolones. Later, the complex chemical structure of vancomycin was ultimately described in 1983 [7]. The genes of OxyB, OxyA and OxyC, encoding three cytochrome P450 enzymes, have been proven to play an important role in three aromatic cross-links of vancomycin in that order [8]. The discovery of X-domain demonstrated the role of OxyA and OxyB, which introduce bisaryl ether linkages with the help of X-domain; however, the mechanism of final crosslink of the biaryl bond installed by OxyC has not been found yet [9]. In 1959, rifamycin was isolated from *Amycolatopsis mediterranei*, which was the first group of antimicrobials targeting RNA polymerase. The genes of RifZ and RifQ were crucial regulatory factors of rifamycin biosynthesis. RifZ directly regulated transcription of all operons within the rifamycin biosynthesis gene cluster [10]. RifQ inhibited the export of rifamycin B and inactivating it could increase the yield of rifamycin B without affecting the growth of the *A. mediterranei* [11]. The understanding of metabolite biosynthesis is helpful to the rational operation of biosynthetic pathways, so as to achieve the goal of producing new natural antibiotics. At present, there are few studies on the biosynthesis of other secondary metabolites of *Amycolatopsis* [12]. We believe that outstanding bioactive compounds from *Amycolatopsis* deserve to be further researched on the mechanism of action, biosynthesis and regulatory genes. Some *Amycolatopsis* species have also been demonstrated to possess great potential in degrading plastics, treating heavy metals, and biotransformation. Herein, we describe a detailed summary about the chemical structures and bioactivities of secondary metabolites from *Amycolatopsis* reported during 1990–2020 by searching in the Web of Science, Google Scholar and PubMed. In addition, the biosynthetic pathways of several potent bioactive compounds and the derivatives of secondary metabolites via chemical synthesis, semi-synthesis and biosynthesis are also described in this paper.

## 2. Secondary Metabolites from the Genus *Amycolatopsis*

Secondary metabolites from *Amycolatopsis* are classified into polyphenols, linear polyketides, macrolides, macrolactams, thiazolyl peptides, cyclic peptides, glycopeptides, amide and amino derivatives, glycoside derivatives, enediyne derivatives and sesquiterpenes, which are all shown in Table 1.

### 2.1. Polyphenols

Polyphenolic compounds are a large family of natural products and some of them show a series of excellent function in health [56], such as anti-allergenic, anti-inflammatory, anti-microbial, antioxidant, antithrombotic, cardio protective, and vasodilatory effects [57]. The investigation on secondary metabolites of *Amycolatopsis* sp. ML630-mF1 from the soil sample collected in Toba of Japan led to the isolation of five new compounds named kigamicins A–E (**1**–**5**). These compounds showed potent effects to resist methicillin-resistant *Staphylococcus aureus* (MRSA) with the IC_50_ values ranging in 0.03–0.22 µM. Besides, they inhibited PANC-1 cell survival under a nutrient-starved condition. Typically, kigamicin D was found to suppress diverse mouse cancer cell line growth, and the IC_50_ value was about 0.95 µM [13]. In the absence of nutrition, kigamicin D exhibited preferential cytotoxicity to cancer cells and could inhibit the PI3K/Akt pathway [58]. A total of 10 novel pentangular polyphenols defined as amexanthomycins A–J (**6**–**15**) were obtained from the fermentation products of *Amycolatopsis mediterranei* S699 ∆rifA (the *A. mediterranei* S699 mutant strain). These compounds were produced through deleting polyketide synthase genes related to rifamycin biosynthesis. In this study, the effects of the above compounds on suppressing topoisomerases IIα (Topo IIα) were examined. The results showed that compounds **6**–**8** exhibited moderate inhibitory activity against Topo IIα (500 μM), while compounds **9**–**15** showed no activities [14].

**Figure 1:**
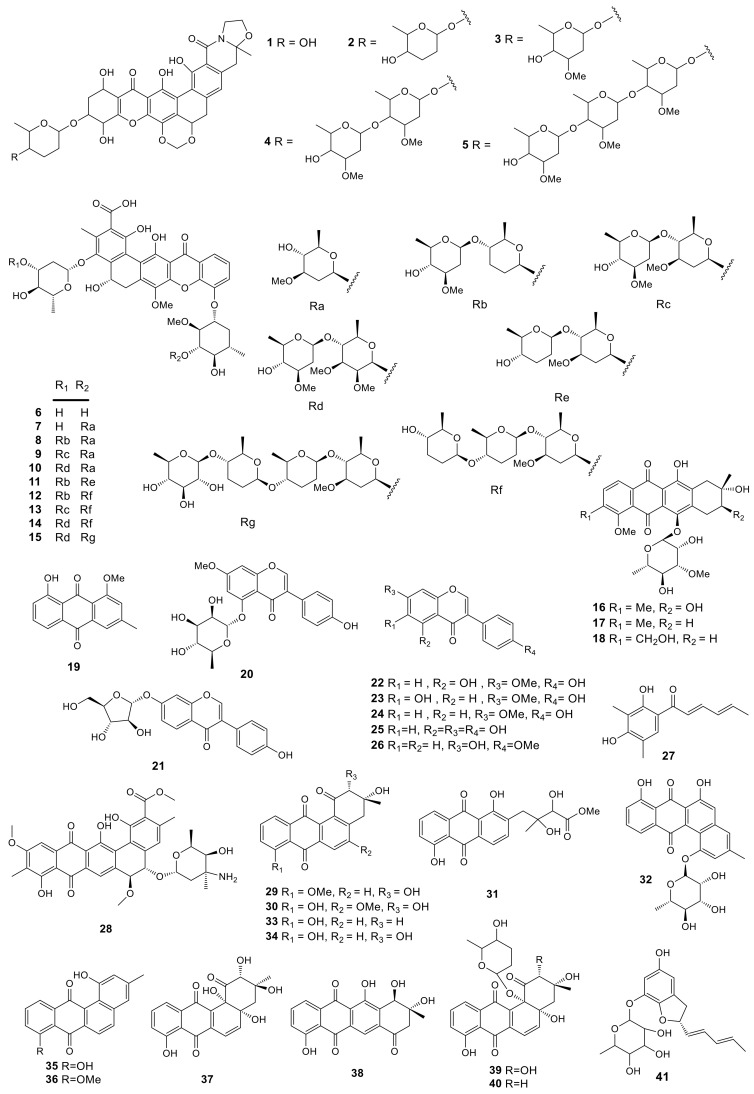
Structures of polyphenols (**1***–***41**) from *Amycolatopsis*.

Anthraquinones are the most abundant among the various natural quinone compounds. Earlier, they were mainly used as dyes. But later, their antibacterial, anti-inflammatory, and antiviral effects were discovered. A new anthracycline, namely, mutactimycin E (**16**) with two known compounds mutactimycin A (**17**) and D (**18**) were isolated from the EtOAc extract of *Amycolatopsis* sp. 17,128 collected from the soil sample near Ruby, Arizona. It had moderate effects to resist some Gram-positive bacteria [15]. Investigation of secondary metabolites from *Amycolatopsis thermoflava* SFMA-103 led to the isolation of the 1-methoxy-3-methyl-8-hydroxy-anthraquinone (**19**) pigment from the rhizosphere soil of sunflower collected in Medak, Andhra Pradesh, South India. Compound **19** displayed infusive anti-cancer activity in-vitro to resist lymphoblastic leukemia as well as lung cancer cells, with the IC_50_ values of 16.98 and 10.3 µM, separately. In addition, the DPPH assay showed that this compound had favorable capacity to scavenge free radicals with the EC_50_ value of 18.2 µg/mL [16]. Furthermore, compound **19** suppressed α-glucosidase and α-amylase with IC_50_ values of 10.32 and 0.91 µM, respectively. According to the research on the oral dose for Wistar rats, compound **19** remarkably suppressed the elevated glucose level at a dose of 100 mg/kg. Its toxicity was further assayed by the genotoxic analysis in both Chinese Hamster Ovary cells (in-vitro) and Swiss albino mice (in-vivo). The studies indicated that compound **19** had little effect on mouse survival. It was concluded that compound **19** was used at 100 mg/kg to treat hyperglycemia via inhibiting α-glucosidase and α-amylase enzymes without inducing any genotoxic effect [17]. 7-*O*-Methyl-5-*O*-α-L-rhamnopyranosylgenestein (**20**) was a novel isoflavonoid glycoside, while 7-*O*-α-D-arabinofuranosyl daidzein (**21**) was firstly extracted from natural sources. These two compounds, along with 5 known isoflavonoids, prunetin (**22**), kakkatin (**23**), isoformononetin (**24**), genistein (**25**), and formononetin (**26**) were produced by the lichen-associated *Amycolatopsis* sp. YIM 130642. Compounds **20** and **21** showed modest bacteriostatic activities against one or more pathogenic strains of *Candida albicans*, *Escherichia coli*, MRSA, *S. aureus*, and *Salmonella typhi* with their minimal inhibition concentrations (MICs) in the range of 32–256 μg/mL [18]. Sorbicillin (**27**) was isolated from the lichen-derived actinomycete strain *Amycolatopsis* sp. YIM 130,687 [19]. Isolation and identification of a new polycyclic antibiotic, pradimicin-IRD (**28**), was reported from the rare actinobacteria *Amycolatopsis* sp. IRD-009, which was collected from soil sample of Brazilian rainforest undergoing restoration area. Compound **28** exhibited antimicrobial activity against *Streptococcus agalactiae*-97, *S. aureus*-211 and *Pseudomonas aeruginosa* ATCC 27.859 with MIC values of 3.15 μg/mL. In addition, the cytotoxicity of compound **28** was determined by MTT assay, which inhibited HCT-116 colon carcinoma, MM 200 melanoma, MCF-7 breast carcinoma and RPE non-tumor retinal pigment epithelial cells with IC_50_ values of 0.8, 2.7, 1.55 and 1.48 μM, respectively [20]. Compound **28** could induce DNA damage (increased γH2AX and p21), cell cycle arrest (reduced Rb phosphorylation) and apoptosis (PARP1 and caspase 3 cleavage). It was capable of impacting on double stranded DNA which might be the novel target for compound **28** [59]. Three new angucyclines, (2*R*,3*R*)-2-hydroxy-8-*O*-methyltetrangomycin (**29**), (2*R*,3*R*)-2-hydroxy-5-*O*-methyltetrangomycin (**30**), amycomycin B (**31**), and a novel angucyclinone derivative, amycomycin A (**32**), with eight known compounds, tetrangomycin (**33**), pd116779 (**34**), tetrangulol (**35**), X-14881E (**36**), sakyomicin B (**37**), tetracyclinone (**38**), sakyomicin A (**39**), and sakyomicin C (**40**), were produced by *Amycolatopsis* sp. Hca1 [21,22], which was collected from the gut of *Oxya chinensis*. Compounds **33**, **34**, **39** and **40** possessed cytotoxic activities against the HeLa cells with the IC_50_ values of 0.27, 0.11, 0.56 and 0.39 μM, respectively, and compound **40** was also cytotoxic against BGC823, HepG2, A375, KB, and Ghost-R5 × 4 cell lines with the IC_50_ values of 11.03, 17.36, 17.5 and 14.0 μM, respectively. Amycofuran (**41**) is a new benzofuran glycoside isolated from *Amycolatopsis saalfeldensis* collected from a sponge sample [23]. All 41 polyphenols described above are presented in Figure 1.

### 2.2. Linear Polyketides

**Figure 2:**
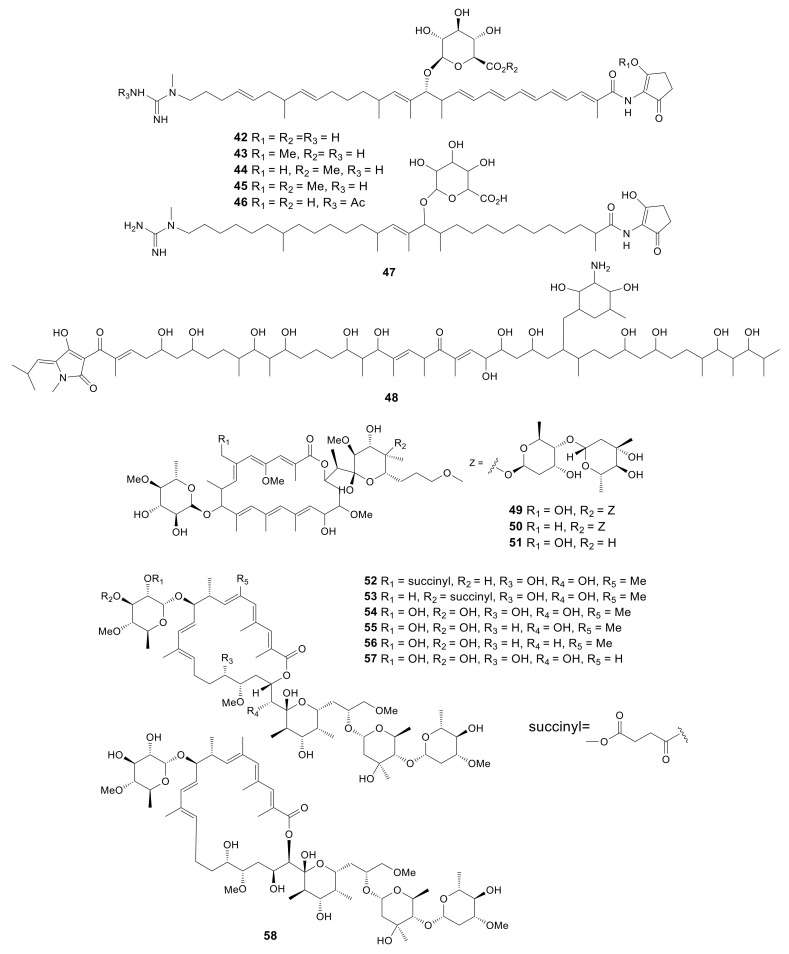
Structures of linear polyketides (**42***–***48**) and macrolides (**49***–***58**) from *Amycolatopsis*.

Through genomic analysis, the strain *Amycolatopsis orientalis* ATCC 43,491 was found to be the producer of vancomycin, which possessed genetic loci to produce over 10 secondary metabolites apart from vancomycin. It was estimated that a gene cluster containing the type I polyketide synthase mediated the biosynthesis for a new glycosidic polyketide ECO-0501 (**42**) [24]. Compound **42** exhibited stronger antibacterial activity than vancomycin against *S. aureus* ATCC TM 6538P in pH 5.0 and 6.0 with the MIC values of 0.125 and 0.25 μg/mL. This compound had potent effect on resisting Gram-positive bacteria MRSA and vancomycin-resistant *Enterococci* (VRE) strains. The mechanistic studies proved that ECO-0501 may impact on either cell wall or membrane biosynthesis [60]. In addition, compound **42** chemical modified analogs, including esterified **43**–**45**, N-acetylated **46**, and hydrogenated **47** were reported. Compound **46** showed antibacterial activity against *S. aureus* ATCC TM 6538P with MIC values of 0.25, 0.5 and 2 μg/mL in pH 5, 6 and 7, respectively. The novel antibiotic vancoresmycin (**48**) was obtained from the culture broth of *Amycolatopsis* sp. ST 101170. It showed a potent effect on resisting the Gram-positive strains of *E. faecium*, *S. aureus*, *S. pneumonia*, *S. epidermidis*, *S. pyogenes*, together with a variety of drug-resistant microorganisms. The IC_50_ values were found to be less than 0.05 μM. By a non-pore forming and concentration-dependent depolarization mechanism, compound **48** selectively targeted the cytoplasmic membrane of gram-positive bacteria [61]. No inhibitory effect against gram-negative bacteria or anti-fungal activity was observed [25]. All 7 linear polyketides described above are presented in Figure 2.

### 2.3. Macrolides

Three novel glycosylated macrolactones, amycolatopsins A–C (**49**–**51**), were produced by *Amycolatopsis* sp. MST-108494 obtained from the soil in southern Australia. Both compounds **49** and **51** prevented *M. tuberculosis* (H37Rv, IC_50_ values of 4.4 and 5.7 μM) and *M. bovis* (BCG; IC_50_ values of 0.4 and 2.7 μM) from growing within the liquid culture. In addition, compounds **49** and **50** showed significant toxicity to the human lung cancer (NCIH-460; IC_50_ values of 1.2 and 0.28 μM) and colon carcinoma (SW620; IC_50_ values of 0.08 and 0.14 μM) cell lines. Whereas, compound **51** showed 5- to 100-fold less cytotoxicity with IC_50_ values of 5.9 and 10 μM, respectively [26]. Two new apoptolidins, 2′-*O*-succinyl-apoptolidin A (**52**) and 3′-*O*-succinyl-apoptolidin A (**53**), with five known compounds, apoptolidins A–D (**54**–**57**) and isoapoptolidin A (**58**), were produced by the Indonesian *Amycolatopsis* sp. ICBB 8242 isolated from the Black Water Ecosystems in Kalimantan. Compound **54** could inhibit the human H292 and HeLa cells with IC_50_ values of 0.02 and 0.04 μM, respectively. Compounds **52** and **53** could suppress the human H292 cell with IC_50_ values of 0.09 and 0.08 μM, respectively [27]. All 10 macrolides described above are presented in Figure 2.

### 2.4. Macrolactams

**Figure 3:**
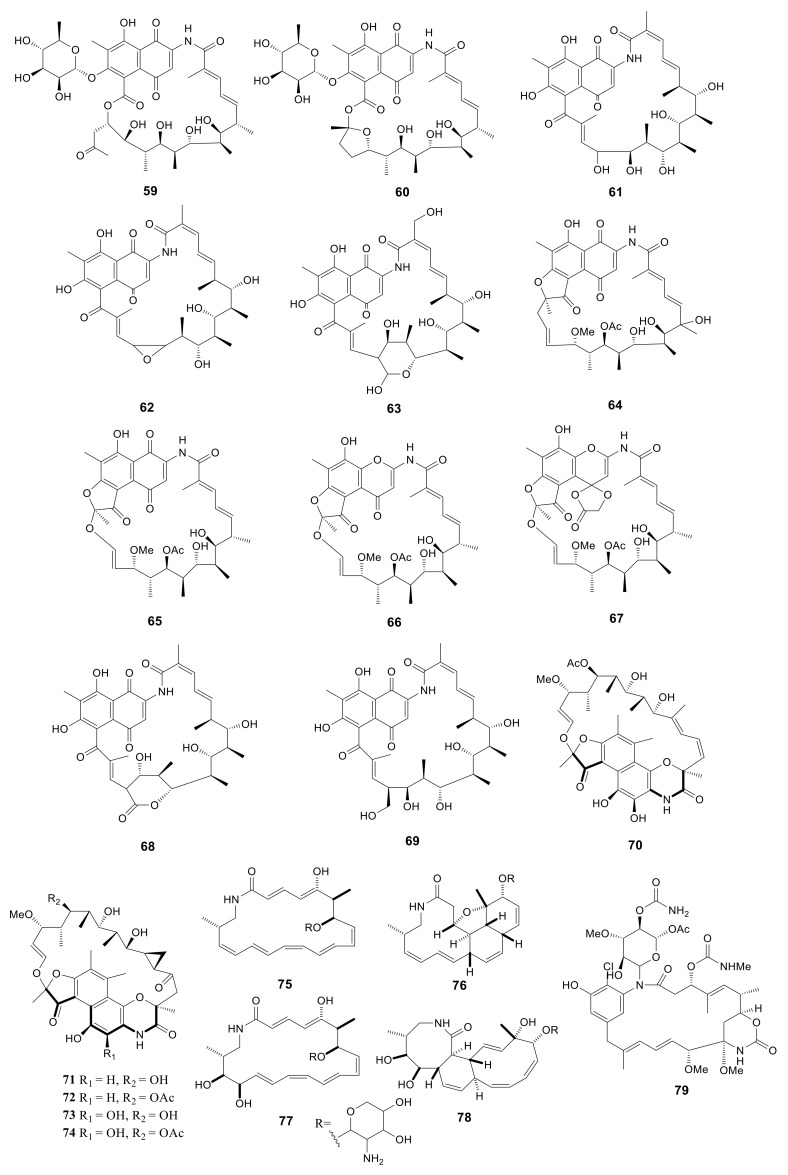
Structures of macrolactams (**59***–***79**) from *Amycolatopsis*.

Macrolactams have been used in clinical trials since 1940 [62], in which penicillin and cephalosporins are the representative antibiotics. For better exploiting the rifamycin diversity, the *Amycolatopsis mediterranei* S699 strain was cultured on the YMG agar media. Eleven rifamycin congeners, including six new compounds, rifamycinosides A (**59**) and B (**60**), 28-desmethyl-28-hydroxyrifamycin W (**61**), 27,28-epoxy-28-desmethylrifamycin W (**62**), 30-hydroxyrifamycin W hemiacetal (**63**) and 20-hydroxyrifamycin S (**64**), with five known compounds, rifamycin S (**65**), 16,17-dehydrorifamycin G (**66**), rifamycins O (**67**), Z (**68**) and W (**69**), were isolated. Compounds **59** and **60** possess the similar skeleton of rifamycin glycosides. The polyketide cores of these two compounds presented the new rifamycin ansa chain cleavage pattern. Compound **64** showed potent inhibitory activity against T3SS, caused G2/M phase arrest, and attracted DNA damage in HCT116 cells [28]. Five unusual macrolactams, rifamorpholines A–E (**70**–**74**), were isolated from *Amycolatopsis* sp. HCa4 collected from the gut of *Locusta migratoria*. Compounds **71** and **73** possessed antimicrobial activity against MRSA, *S. aureus*, *S. pyogenes*, *Bacillus subtilis*, and *Micrococcus luteus* with MIC values in the range of 0.5–8.0 Μm [29]. Four new 20-membered glycosylated polyketide macrolactams, macrotermycins A–D (**75**–**78**), were produced by *Amycolatopsis* sp. M39 collected from a *Macrotermes natalensis*. Compound **75** exhibited antimicrobial activity against *B. subtilis* ATCC 6051, *S. aureus* ATCC 25923, *Saccharomyces cerevisiae* ATCC 9763 and *C. albicans* ATCC 24,433 with MIC values of 1.0, 1.5, 5.0, 10, respectively. And compound **77** exhibited antimicrobial activity against *B. subtilis* ATCC 6051, *S. aureus* ATCC 25923, *Saccharomyces cerevisiae* ATCC 9763 and *C. albicans* ATCC 24,433 with MIC values of 15, 10, 20, 25 μg/mL, respectively [30]. A novel compound, ansamycin (**79**), was produced by *Amycolatopsis alba* DSM 44262. However, this compound exhibited no antimicrobial activity for *S. aureus*, *B. subtilis*, *P. aeruginosa* and *C. albicans* [31]. All 21 macrolactams described above are presented in Figure 3.

### 2.5. Thiazolyl Peptides

**Figure 4:**
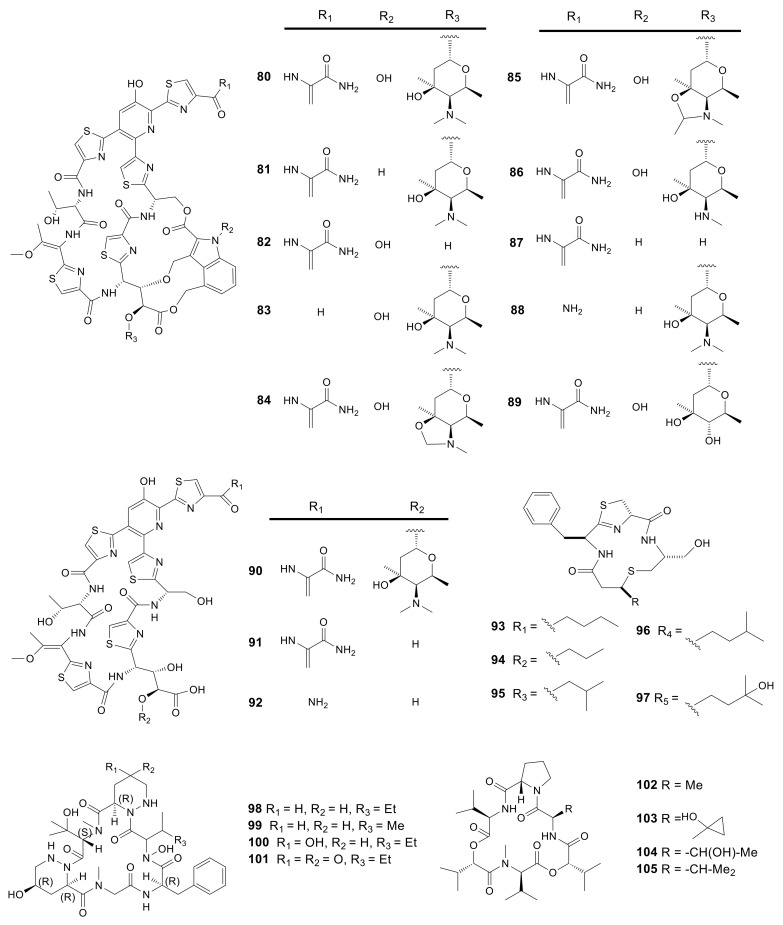
Structures of thiazolyl peptides (**80**–**97**) and cyclic peptides (**98**–**105**) from *Amycolatopsis*.

After chromatographically fractionating the fermentation broth extract of *Amycolatopsis fastidiosa*, 4 known nocathiacins I–IV (**80**–**83**) along with 2 novel thiazolyl peptides, thiazomycin (**84**) and thiazomycin A (**85**) were isolated [32,33,34]. Compounds **84** and **85** showed potent inhibition against Gram-positive bacteria. Continued chemical screening led to the separation of an intermediate product and six new thiazolyl peptide congeners, MJ347-81F4 B (**86)**, thiazomycins B–D (**87**–**89**), and E_1_-E_3_ (**90**–**92**). The new compounds were tested for their antimicrobial activity against gram-positive bacterial strains of *S. aureus*, *E. faecalis*, *S. pneumonia*, and other drug-resistant strains. The results indicated that compounds **87**–**89** effectively inhibited the growth of the pathogenic bacteria described above, whereas compounds **90**–**92** showed no obvious antimicrobial activity [35]. Five novel compounds thioamycolamides A–E (**93**–**97**) were obtained from the fermentation products of *Amycolatopsis* sp. 26−4 isolated from Iriomote Island near Okinawa, Japan. They were cycliclipopeptides containing sulfur, thioether rings, thiazoline, along with fatty acid moieties. Compounds **93** and **96** showed moderate cytotoxicity with the IC_50_ values ranging from 6.53 to 21.22 μM. However, compound **97** had an IC_50_ value greater than 100 μM [36]. All 18 thiazolyl peptides described above are presented in Figure 4.

### 2.6. Cyclic Peptides

Cyclic peptides always possess antibacterial, antitumor, hypotoxic, immunosuppressive activities and have a merit of favorable binding affinity and selectivity for certain receptors [63]. The limited conformational freedom conferred by cyclization enables cyclic peptides to span large surfaces while retaining the conformational restriction that yields high selectivity and affinity. Such advantages render them the ideal selection for developing therapeutics [64]. While screening antibiotics against MRSA and VRE, the novel cyclic peptide, PRG-A (**98**) containing the distinct piperazic acid, was obtained from the fermentation broth of *Amycolatopsis* sp. ML1-hF4 isolated from a soil sample collected at Shinagawa, Tokyo, Japan [37]. During the optimization of the production process of PRG-A, three new derivatives, namely, PRG-B (**99**), C (**100**), and D (**101**), were further isolated from the same strain. This study examined the effects of these new PRGs on resisting a variety of Gram-positive and -negative bacteria, like VRE and MRSA. The results showed that compounds **98** and **100** exhibited potent and broad antibacterial activity against gram-positive bacteria with the IC_50_ value of about 0.72 μM. The antibacterial activity of compounds **99** and **101** was lower, with the IC_50_ values ranging from 5.61 to 23.37 μM. However, all compounds failed to show anti-bacterial activity against gram-negative bacteria [38]. Compound **98** disrupted cell membrane function by disruption of membrane potential [65]. Another four new cyclic depsipeptide compounds, named valgamicins A (**102**), C (**103**), T (**104**) and V (**105**), were isolated from *Amycolatopsis* sp. ML1-hF4. Compound **105** possessed an excellent cytotoxicity against a series of human tumor cell lines, such as MIA Paca 2 (Pancreatic cancer), HGC-27 (Gastric cancer), GSS (Gastric cancer), 5637 (Bladder cancer), NCI-H1650 (Lung cancer), GI-1 (Glioma), NB16 (Neuroblastoma), ME-180 (Cervical cancer), and HSC-490 (Tongue cancer), with IC_50_ values from 6.6 to 21.6 μM [39]. All 8 cyclic peptides described above are presented in Figure 4.

### 2.7. Glycopeptides

**Figure 5:**
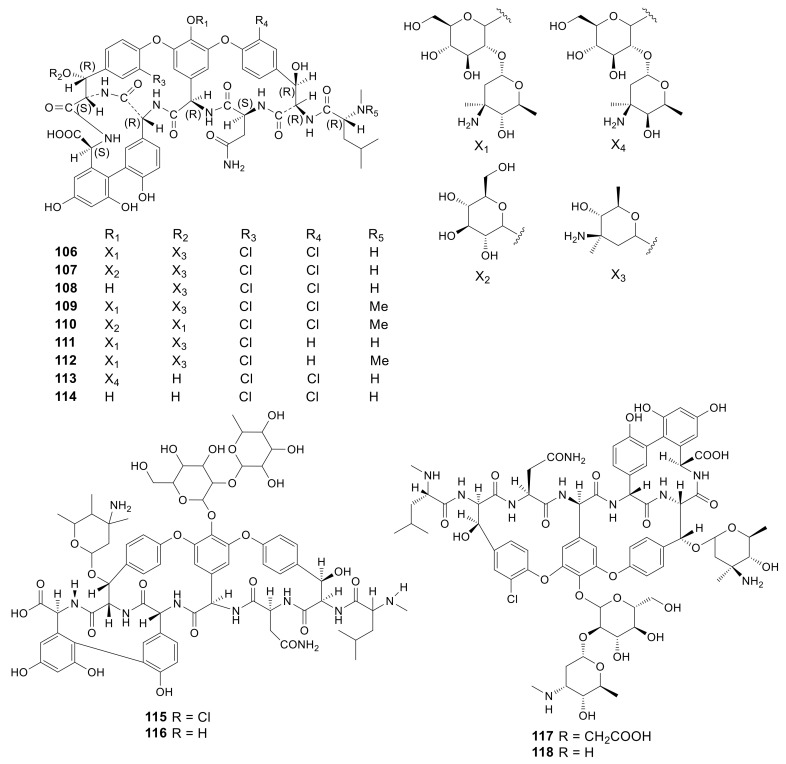
Structures of glycopeptides (**106***–***118**) from *Amycolatopsis*.

Glycopeptide antibiotics are used as a key weapon in against bacteria, especially multidrug-resistant Gram-positive pathogens. The ground-breaking work about glycopeptide antibiotics resistance mechanisms in Gram-positive pathogens were published in the 1990s [66]. Chloroorienticins were similar to vancomycin-type antibiotics. Five new chloroorienticins A–E (**106**–**110**), orienticins A (**111**) and D (**112**), vancomycin (**113**) and its aglycone (**114**) were isolated from the fermentation broth of *Amycolatopsis orientalis* PA-45052. Some of them showed higher antibacterial activity than vancomycin. Compounds **106**–**110** showed significant antibacterial activity against *S. aureus* JC-1 and MRSA with MIC values in the range of 0.2–0.78 μg/mL. Vancomycin (**113**) was comparatively against these two bacteria with MIC values of 0.78 and 1.58 μg/mL, respectively [40]. MM 47,761 (**115**) and MM 49,721 (**116**) were obtained from *Amycolatopsis orientalis* NCBI 12,608 and displayed a favorable antimicrobial effect on Gram-positive strains. Compounds **115** and **116** could inhibit *B. subtilis* ATCC6633, *Corynebacterium xerosis* NCTC9755, *M. luteus* NCTC8340, *S. aureus* Oxford, *S. aureus* Russell, *S. aureus* V573 MRa, *S. saprophyticus* FL1, *S. epidermidis* 60137, *S. epidermidis* 54815, *Streptococcus pyogenes* CN10, *S. agalactiae* Hester, *S. sanguis* ATCC 10556, *S. faecalis* I with the MIC values from 0.5 to 8 μg/mL [41]. A new bioactive antibiotic, eremomycin B (**117**), along with one known antibiotic, eremomycin (**118**), were isolated from the culture broth of *Amycolatopsis orientalis* subsp. *Eremomycini* [42]. All 13 glycopeptides described above are presented in Figure 5.

### 2.8. Amide Derivatives

Albachelin (**119**), a novel siderophore, was obtained from the *Amycolatopsis alba* culture with iron depletion. Then, ESI–MS/MS together with NMR spectroscopy was performed to characterize the gallium (III) complex [43]. Albisporachelin (**120**), a new siderophore, was obtained from the *Amycolatopsis albispora* WP1^T^ culture broth with iron depletion using sediments obtained at −2945 m from the Indian Ocean [44]. In the course of bacterial translocase I inhibitor screening, a new compound, A-102395 (**121**) was isolated from *Amycolatopsis* sp. SANK 60206. A-102395 (isolated from a soil sample collected in Hokkaido, Japan) showed strong inhibition on the bacterial translocase I with the IC_50_ value of 0.01 μM. This compound showed no antibacterial effect on the analyzed strains [45]. The indole alkaloids are associated with cyclopiazonic acids, which were previously only detected in fungi. In addition, amycocyclopiazonic acid (**122**), along with amycolactam (**123**), was obtained from *Amycolatopsis saalfeldensis*. Combined with spectroscopic data, the structures of compounds **122** and **123** were identified to be new indole alkaloids related to cyclopiazonic acids. Amycolactam was significantly cytotoxic to gastric cancer SNU638 and colon cancer HCT116 cells, and the IC_50_ values were 0.8 and 2.0 μM, respectively [23]. The novel derivative of carbamothioic S-acid (**124**) was obtained from *Amycolatopsis alba* DSM 44262∆abm9 fermentation extract exposed to 25 mM N-acetyl-D-glucosamine [46]. Amycophthalazinone A (**125**), the first example of natural occurring new phthalazinone derivative, was discovered from the fermentation products of the lichen-associated *Amycolatopsis* sp. YIM 130,642 [18]. Compound **125** had potent antibacterial effect on *S. typhi*, *C. albicans*, and *S. aureus*, with IC_50_ values of 6.92, 13.84, and 6.92 μM, respectively. 2-Pyruvoylaminobenzamide (**126**), (–)-chrysogine (**127**), 4-(3-methylbut-2-enyloxy) benzamide (**128**), acetotryptamide (**129**), 2-acetamidophenol (**130**), anthranilic acid (**131**), phenacetamide (**132**) and 2-carbamoyl-3-hydroxy-1,4-naphthoquinone (**133**) were isolated from the cultural of *Amycolatopsis* sp. YIM 130687. Compounds **128** and **133** were firstly discovered from microorganisms [19].

**Figure 6:**
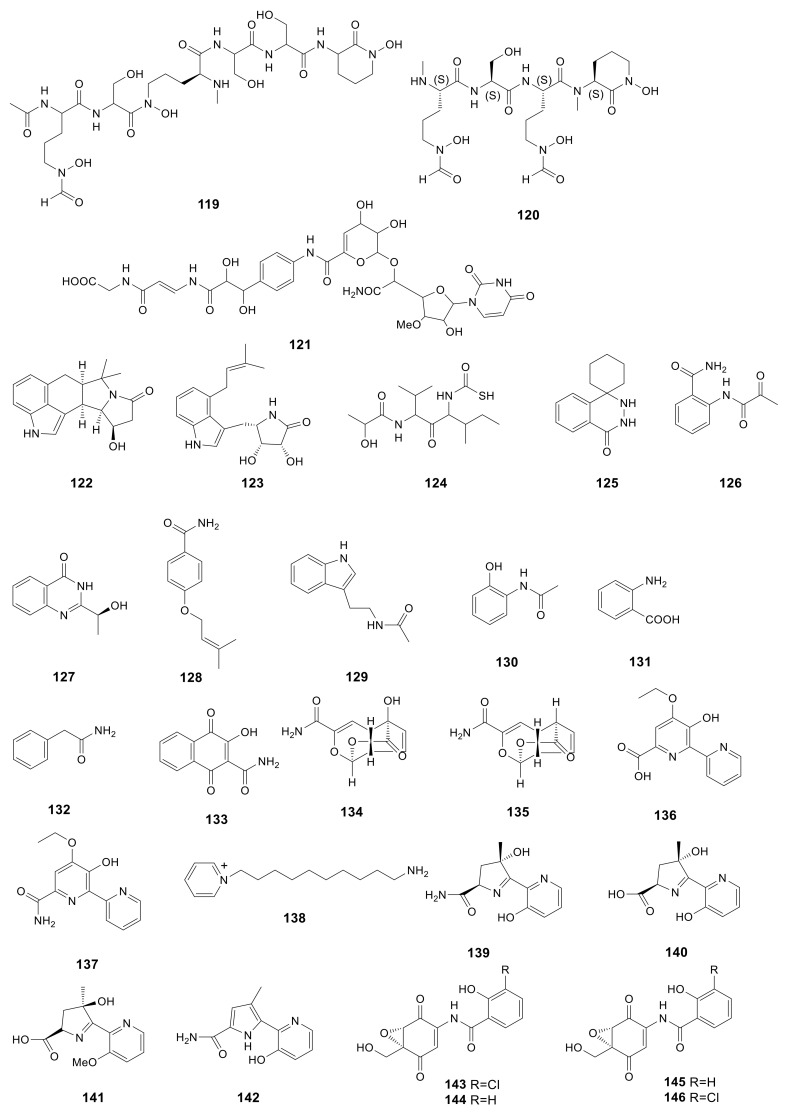
Structures of amide derivatives (**119***–***146**) from *Amycolatopsis*.

Two novel echinosporin derivatives, echinosporin (**134**) and 7-deoxyechinosporin (**135**), were obtained from the culture broth of *Amycolatopsis* sp. YIM PH20520 from the *Panax notoginseng* rhizosphere soil samples collected from Wenshang, Yunnan Province of China. Compound **134** had potent effect on resisting four *P. notoginseng* root-rot pathogens, including *Fusarium solani*, *Fusarium oxysporum*, *Phoma herbarum* and *Alternaria panax*, and the MIC values were 64, 64, 64 and 32 μg/mL, respectively. Compound **135** had moderate effect on resisting *F. solani*, *F. oxysporum*, *P. herbarum* and *A. panax*, with the MIC values of 128, 128, 128 and 64 μg/mL, respectively [47]. Two novel compounds, dipyrimicins A (**136**) and B (**137**), were produced by *Amycolatopsis* sp. K16-0194. Compound **136** exhibited excellent antimicrobial activity against *S. cerevisiae* ATCC 9763, *Kocuria rhizophila* ATCC 9341, *B. subtilis* ATCC 6633, *E. coli* NIHJ, *Xanthomonas campestris* pv. oryzae KB 88 with the inhibition zone from 16 to 21 mm in a dose of 30 μg and from 11 to 27 mm in a dose of 100 μg. Compound **136** also displayed strong cytotoxic activity against Hela 3S, HT29, A549, H1299, Panc1, THP-1, Jarkat and HL-60 with the IC_50_ values of 5.1 ± 0.5, 6.2 ± 0.3, 4.3 ± 0.2, 9.2 ± 0.5, 9.4 ± 3.5, 9.4 ± 3.5, 4.4 ± 0.5 and 3.9 ± 0.7 μM, respectively. Compound **137** only had a moderate inhibition on H1299 cell line with an IC_50_ value of 6.8 ± 3.3 μM [48]. A new pyridinium, 1-(10-aminodecyl) pyridinium (**138**), was produced by *Amycolatopsis alba* var. nov. DVR D4, which was collected from marine sediment of Visakhapatnam coast. With a dose of 1000 μg/mL, compound **138** had a great effect on HeLa, MCF-7 (breast cancer), U87MG (brain cancer) cells with percentage viability (%) and percentage inhibition (%) of 39.54, 60.36, 58.15 and 60.46, 39.64, 41.85, respectively [49]. Three novel siderochelins D–F (**140**–**142**), with the known siderochelin A (**139**) were obtained from *Amycolatopsis* sp. LZ149, derived from the rhizosphere of *Cynodon dactyIon* in the Baicheng beach of Xiamen, Fujian, China. Compound **139** exhibited antimicrobial activity against *Bacillus pumilus* CMCC55051, *B. subtilis* CMCC63501, *E. coli* CMCC4103 and *S. aureus* CMCC2600 with the diameter of inhibition zone from 10 to 15 mm [50]. Epoxyquinomicins A–D (**143**–**146**), four new compounds were isolated from the cluture broth of *A. sulphurea* MK299-95F4 from the soil sample collected at Sendai City, Miyagi Prefecture, Japan. Compounds **143** and **144** exhibited antimicrobial activity against *M. luteus* IFO3333 and *M. luteus* PCI1001 with MIC values from 3.12 to 6.25 μg/mL [51]. Compounds **143**–**146** (1–4 mg/kg) possessed an inhibition ability of type II collagen-induced arthritis [67]. All 28 amide and amino derivatives described above are presented in Figure 6.

### 2.9. Glycoside Derivatives

**Figure 7:**
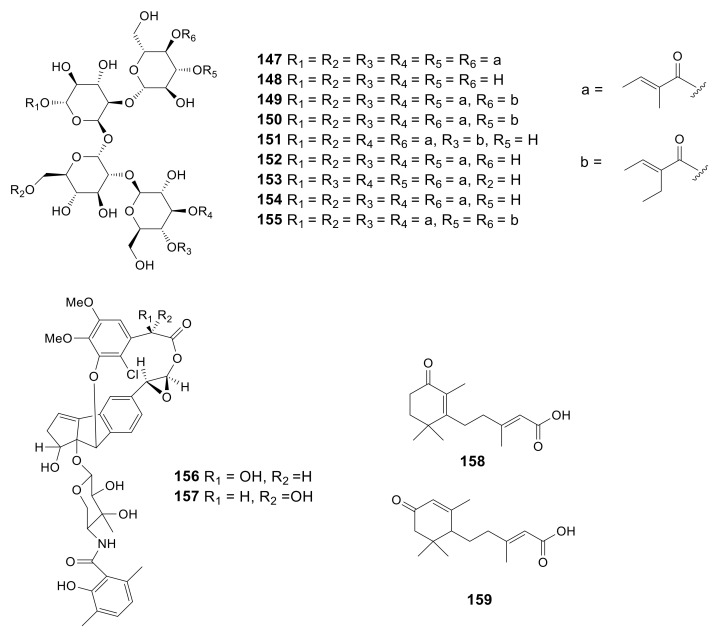
Structures of glycoside derivatives (**147***–***155**), enediyne derivatives (**156***–***157**) and sesquiterpenes (**158***–***159**) from *Amycolatopsis*.

HPLC-diode array screening was used to isolate tigloside (**147**) and 2,2′-di-*O*-β-D-glucopyranosyl-α-D-glucopyranosyl α-D-glucopyranoside (**148**) from the *Amycolatopsis* sp. NN0 21,702 mycelium. Chromatographic approaches were used to purify these new compounds, while NMR spectroscopy together with chemical degradation assays was adopted to confirm their structures [52]. Three new tetrasaccharide derivatives, actinotetraoses I–K (**149**–**151**), with three known compounds, actinotetraoses A–C (**152**–**154**), were isolated from *Amycolatopsis* sp. HCa1, which was collected from the gut of grasshopper [53]. Another novel tetrasaccharide derivative, actinotetraose L (**155**), was also obtained from the *Amycolatopsis* sp. HCa1 [54]. However, these compounds showed no significant bioactivity. The 9 glycoside derivatives described above are presented in Figure 7.

### 2.10. Enediyne Derivatives

Amycolamycins A (**156**) and B (**157**), two new enediyne derivatives, were isolated from *Amycolatopsis* sp. HCa4, which was collected from locust. Compound **156** could inhibit M231 cell lines by inducing apoptosis through activation of caspase-3 with the IC_50_ value of 7.9 μM [55]. The two enediyne derivatives described above are presented in Figure 7.

### 2.11. Sesquiterpenes

Two novel abscisic acid-type sesquiterpenes, (*E*)-3-methyl-5-(2,6,6-trimethyl-3-oxocyclohex-1-enyl)pent-2-enoic acid (**158**) and (*E*)-3-methyl-5-(2,6,6-trimethyl-4-oxocyclohex-2-enyl)pent-2-enoic acid (**159**), were produced by *Amycolatopsis alba* DSM 44,262 [31]. However, these two compounds exhibited no antimicrobial activity against *S. aureus*, *B. subtilis*, *P. aeruginosa* and *C. albicans*. The two sesquiterpenes described above are presented in Figure 7.

## 3. Biofunction of *Amycolatopsis* Species

The *Amycolatopsis* species have potential for biological degradation, bioconversion and biosorption, which might solve the problem of environmental pollution in the future [68].

### 3.1. Biological Degradation

ZJ0273 was a widely used broad-spectrum herbicide and left in soil in large numbers. Cai et al. found that ZJ0273 could be utilized by *Amycolatopsis* sp. M3-1 as the sole carbon and energy source with higher degrading activity. At 30 °C and pH 7.0, the efficiency of ZJ0273 degradation by *Amycolatopsis* sp. M3-1 was 59.3% and 68.5% in 25 and 60 days, respectively [69]. Naproxen was a drug utilized by humans; however, it was detected in surface waters and sanitary effluents in 71 countries, causing toxic effects on biota and further destroying the ecological environment [70]. At the concentration of 50 mg/L, naproxen could be used as the sole carbon and energy source of *Amycolatopsis* sp. Poz 14 and completely degraded in 18 days, while it will affect the growth of *Amycolatopsis* when its concentration was more than 50 mg/L [71]. It takes a long time for plastics to degrade in nature and the resulting environmental pollution problems are becoming more and more serious. Some *Amycolatopsis* strains possessed a polylactic acid (PLA) degradation capability including *Amycolatopsis* sp. HT-32, *Amycolatopsis* sp. 3118, *Amycolatopsis* sp. KT-s-9, *A. mediterranei* ATCC 27649, *Amycolatopsis* sp. 41, *Amycolatopsis* sp. K104-1, *A. orientalis* ssp. *orientalis*, *Amycolatopsis thailandensis* CMU-PLA07T and *Amycolatopsis* sp. SCM_MK2-4 [72]. Tan et al. proved that *A. mediteranei* was capable to hydrolyze the aliphatic plastics poly(ε-caprolactone) and poly(1,4-butylene succinate) via a extracellular lipase [73]. In addition, *Amycolatopsis* sp. ATCC 39,116 could depolymerize high molecular weight lignin species and catabolize a significant portion of the low molecular weight aromatics and may become a mature route for biological lignin valorization in the future [74].

### 3.2. Bioconversion

The strains of *Amycolatopsis* sp. HR167 and *Amycolatopsis* sp. ATCC39116 were able to convert ferulic acid (cell wall component of higher plants) into vanillin (important flavor compound) with concentrations of 11.5 and 13.9 g/L, respectively. The vanillin production of vdh (encoded vanillin dehydrogenase) mutant of *Amycolatopsis* sp. ATCC39116 was increased 2.3 times due to the enzyme catalyzed the catabolism of vanillin [75]. Wuxistatin, a novel HMG–CoA reductase inhibitor, was transformed from lovastatin by hydroxylase (cytochrome P450) and isomerases of *Amycolatopsis* sp. CGMCC 1149, showing a four-fold activity, more than lovastatin [76,77].

### 3.3. Biosorption

Albarracín et al. discovered that *A. tucumanensis* DSM 45,259 (initially named as *Amycolatopsis* sp. AB0) possessed copper specific biosorption ability (25 mg/g) [78,79]. In the presence of Cu(II), *A. tucumanensis* DSM 45,259 enhanced the ability of reducing Cr(VI) [80]. The bioemulsifiers produced by *A. tucumanensis* DSM 45,259 was able to mediate two times Cr(VI) recovery compared to deionized water from soil and maybe utilized to recover Cr(VI) in the future [81]. Baz et al. collected *Amycolatopsis* sp. GT6, *Amycolatopsis* sp. GT15 and *Amycolatopsis* sp. GT39 from abandoned mining areas, which could tolerate high concentrations of metals (Cu, 0.1; Zn, 0.1; Cr, 0.15; Pb, 0.25 mg/mL) [82].

## 4. Bioactivities of Secondary Metabolites from *Amycolatopsis*

The bioactivities of secondary metabolites from *Amycolatopsis* strains have been also presented in Table 2, including antimicrobial, cytotoxic, antioxidant, topo IIα inhibition, anti-hyperglycemic, enzyme inhibition and DNA damage.

Among the total of 159 secondary metabolites, 41 compounds exhibited potent antimicrobial activities, a majority of which showed inhibition on Gram-positive bacteria growth. Most of them were also found to be active against various multi-drug resistant strains. Kigamicins (**1**–**5**), mutactimycin E (**16**), pradimicin-IRD (**28**), ECO-0501 (**42**), vancoresmycin (**48**), amycolatopsins A (**49**) and C (**51**), rifamorpholines B (**71**) and D (**73**), macrotermycins A (**75**) and C (**77**), thiazomycin (**84**), thiazomycins A–D (**85, 87**–**89**), PRG-A–D (**98**–**101**), chloroorienticins A–E (**106**–**110**), vancomycin (**113**), MM 47,761 (**115**), MM 49,721 (**116**) and amycophthalazinone A (**125**) showed significant inhibitory effects against gram-positive bacteria and their drug-resistant types with MIC and IC_50_ values less than 1 μg/mL and 25 μM, respectively. While epoxyquinomicins A (**143**) and B (**144**) displayed moderate activities with MIC values in the range of 3.12–6.25 μg/mL. 7-O-Methyl-5-O-α-L-rhamnopyranosylgenestein (**20**), 7-O-α-D-arabinofuranosyl daidzein (**21**), echinosporin (**134**) and 7-deoxyechinosporin (**135**) showed modest antibacterial activities with MIC values in the range of 32–256 μg/mL.

Of these reported substances, a total of 18 compounds had strong cytotoxicities to different cancer cell lines. For instance, kigamicin D (**4**) suppressed mouse cancer cell growth, and the IC_50_ value was approximately 0.95 μM. 1-Methoxy-3-methyl-8-hydroxy-anthraquinone (**19**) displayed infusive anti-cancer effect on lymphoblastic leukemia together with lung cancer cells, and the IC_50_ values were 16.98 and 10.3 µM, respectively. Pradimicin-IRD (**28**) showed excellent cytotoxicity to HCT-116, MM 200, MCF-7 and RPE with the IC_50_ values of 0.8, 2.7, 1.55 and 1.48 μM, respectively. Tetrangomycin (**33**), pd116779 (**34**), sakyomicins A (**39**) and C (**40**) could inhibit the Hela cells with the IC_50_ values of 0.27, 0.11,0.56 and 0.39 μM, respectively. Amycolatopsins A (**49**) and B (**50**) had potent effects on resisting human colon cancer (SW620; IC_50_ values, 0.08 and 0.14 μM) as well as lung cancer (NCIH-460; IC_50_ values, 1.2, and 0.28 μM) cell lines. 3′-O-Succinyl-apoptolidin A (**52**), 2′-O-succinyl-apoptolidin A (**53**) and apoptolidins A (**54**) could inhibit the H292 cells with the IC_50_ values of 91, 82 and 22 μM, respectively. Thioamycolamides A (**93**) and D (**96**) showed moderate cytotoxicity to fibrosarcoma HT1080 and cervix adenocarcinoma HeLa with the IC_50_ values ranging from 6.53 to 21.22 μM. Valgamicins V (**105**) could inhibit the cancer cells, such as MIA Paca 2, HGC-27, GSS, 5637, NCI-H1650, NB16, ME-180, HSC-490 (IC_50_, 6.6-21.6 μM). Amycolactam (**123**) had marked effect on resisting gastric cancer SNU638 cells as well as colon cancer HCT116 cells. The IC_50_ values were recorded to be 0.8 and 2.0 μM, respectively. Dipyrimicin A (**136**) exhibited moderate cytotoxicity to a series of cancer cells (Hela 3S, HT29, A549, H1299, Panc1, THP-1, Jarkat, HL-60) with the IC_50_ from 3.9 to 9.4 μM. However, dipyrimicin B (**137**) only suppressed the H1299 with the IC_50_ value of 6.8 ± 3.3 μM. Amycolamycin A (**156**) showed moderate cytotoxicity to M321 with the IC_50_ value of 7.9 μM.

A total of 15 compounds showed other bioactivities. Amexanthomycins A–J (**6**–**15**) possessed a xanthone-containing pentangular polyphenol core. Compounds **9**–**15** had no inhibitory effect on DNA topoisomerase IIα (Topo IIα), while compounds **6**–**8** exhibited moderate inhibitory activity against Topo IIα at 500 μM. These results showed that the different numbers and types of deoxysugars in compounds **6**–**15** will affect the inhibitory activity of topoisomerase. Compound **19** was used at 100 mg/kg to treat hyperglycemia without inducing any genotoxic effect and also inhibiting α-amylase and α-glucosidase with the IC_50_ values of 0.91 and 10.32 µM, respectively. Experiments in mice have proven the safety and efficacy of compound **19**. Compounds **59**–**61**, **63** and **67** exhibited strong activity for inhibiting Topo I at 50 and 100 μM. Moreover, compounds **59**–**61**, **63**, **65**, **67** and **68** showed the activity of inhibiting Topo IIα at 50 μM. Compound **64** had strong effect on inhibiting T3SS, resulted in cell cycle arrest at G2/M phase, and led to DNA damage within the HCT116 cells. A-102395 (**121**) was identified as the strong bacterial translocase I inhibitor, and its IC_50_ value was 0.011 μM. At the dose of 1–4 mg/kg, epoxyquinomicins C (**145**) and D (**146**) could inhibit type II collagen-induced arthritis.

## 5. Synthesis of Secondary Metabolites from *Amycolatopsis* and Their Derivatives

### 5.1. Biosynthetic Pathways of Secondary Metabolites from Amycolatopsis

Research on biosynthetic pathways is essential for the further study on secondary metabolites. For example, finding the regulatory gene could increase or decrease the production of metabolites and also uncover how the concerted efforts of various enzymes to form the compound [83]. In this review, we list the hypothetical biosynthetic pathways for several potent bioactive compounds. Few studies have been conducted and need to arouse the attention of researchers.

**Figure 8:**
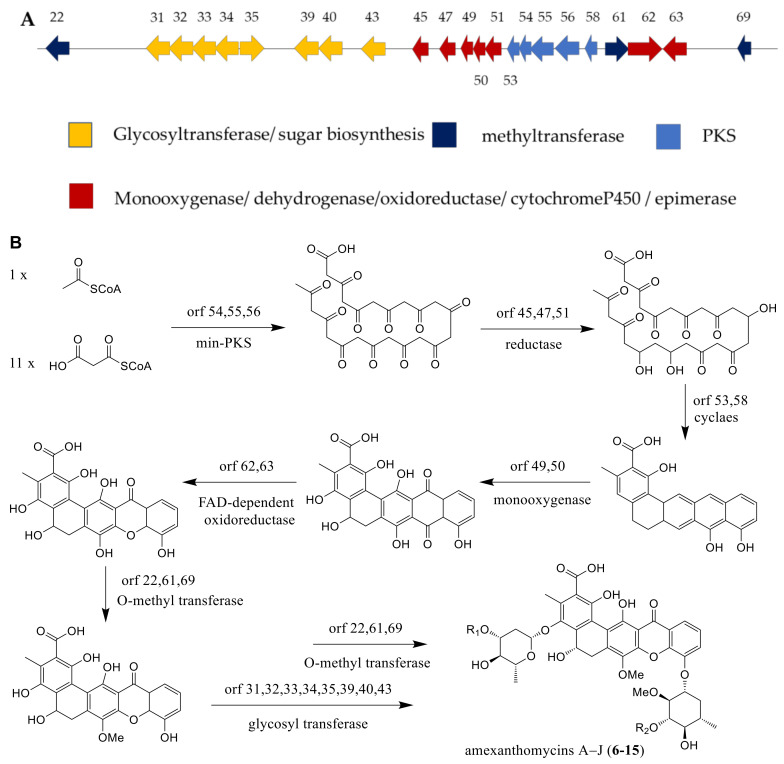
(**A**) The gene clusters of amexanthomycins A–J. (**B**) The biosynthetic pathway of amexanthomycins A–J [14].

The mutant strain *A. mediterranei* S699Δ*rifA*, which was deleted for the biosynthesis gene of rifamycins, displayed the ability for producing ten new pentangular polyphenols, amexanthomycins A–J (**6**–**15**) [14]. As described in the literature, the production of associated genes included polyketide synthase (PKS), glycosyltransferase, methyltransferase, monooxygenase, dehydrogenase, oxidoreductase, cytochrome P450 and epimerase (Figure 8A). The biosynthetic pathway of amexanthomycins were proposed by Li et al. [14] and exhibited in the Figure 8B. An acetyl-CoA starter unit and 11 malonyl-CoA extender units could produce prediction intermediate, the pentacyclic xanthone core, by min-PKS synthase, cyclase, and oxidoreductase. Then, the predicted oxidase catalyzed the oxidative rearrangement reaction of intermediate. Finally, this aglycone was glycosylated by the glycosyl transferases, completing the biosynthesis of compounds **6**–**15** (Figure 8B) [14].

**Figure 9:**
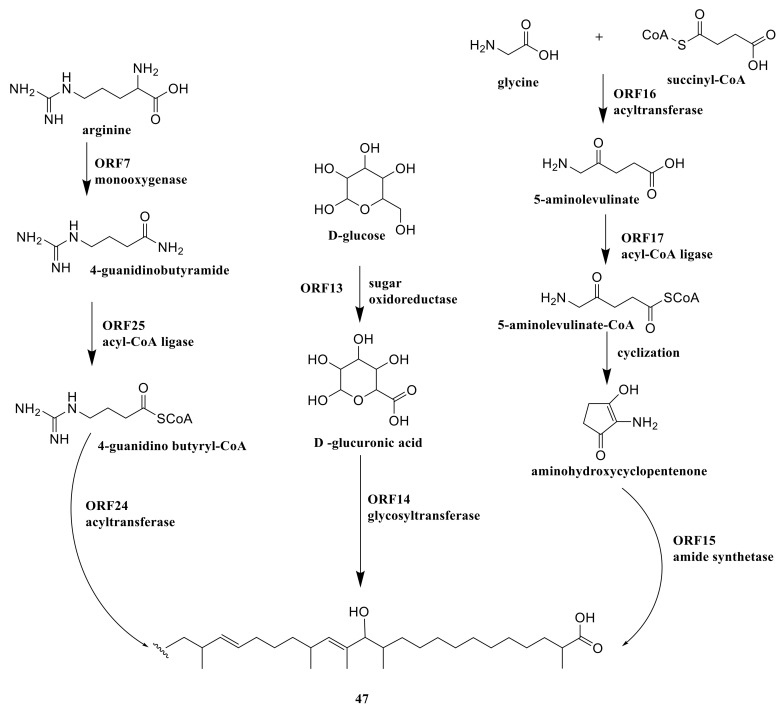
The biosynthetic pathway of ECO-0501 (**47**) [24].

The genome of *A. orientalis* ATCC 43,491 included a type I PKS which encoded by ORF 18-23 and synthesized the polyketide chain [24]. The monooxygenase and acyl-CoA ligase were encoded by ORF 7 and 25 which catalyzed arginine to 4-guanidino butyryl-CoA. D-glucose was catalyzed by oxidoreductase (ORF 13) and turned into D-glucuronic acid. Glycine and succinyl-CoA were transformed into 5-aminolevulinate by acyltransferase (ORF 16), and then turned into 5-aminolevulinate-CoA by acyl-CoA ligase (ORF 17). 5-aminolevulinate-CoA was transformed into aminohydroxycyclopentenone through cyclization reaction by the coenzyme A ester. Three ORFs (14, 15 and 24) provided glycosyltransferase, amide synthetase and acyltransferase to add 4-guanidino butyryl-CoA, D-glucuronic acid and aminohydroxycyclopentenone onto the polyketide chain which formed compound **47** (Figure 9) [24].

**Figure 10:**
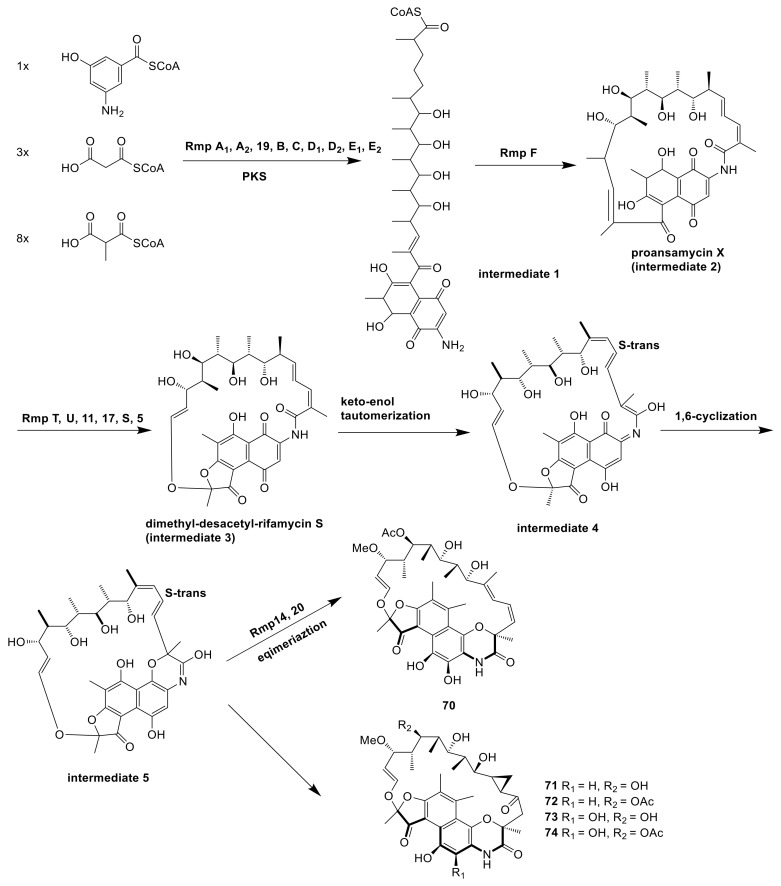
The biosynthetic pathway of rifamorpholines A–E [29].

The genome of *Amycolatopsis* sp. HCa4 was analyzed by antiSMASH and 2ndfind, the cluster 19 was highly similar to the biosynthetic gene cluster of rifamycin [29]. A 3-amino-5-hydroxybenzoic acid starter unit and two malonyl CoA and eight methyl malonyl CoA extender units could produce intermediate 1 on a type I polyketide synthase. The release of the polyketide chain and the formation of intramolecular amide were catalyzed by the amide synthase encoded by Rmp F and then generated proansamycin X (intermediate 2). Proansamycin X was then catalyzed by a serious of enzymes encoded by Rmp T, U, 11, 5, etc., and turned into the key intermediate 3, dimethyl-desacetyl-rifamycin S. All the above synthetic processes were the same as the synthesis of rifamycin, but Xiao et al. did not find the rifamycin analogs in this strain and they suspected that maybe an unidentified enzyme catalyzed the keto–enol tautomerization of intermediate 3 to form intermediate 4. The intermediate 4 was formed to the intermediate 5 through a crucial 1,6-cyclization, which was further converted into compound **70** and compounds **71**–**74** followed by two branch pathways. The formation of compound **70** was catalyzed by 25-O-acetyltransferase (Rmp 20) and C-27-O-methyltransferase (Rmp 14), as well as epimerization of C-21. However, the formation of compounds **71***–***74** was not speculated (Figure 10) [29].

**Figure 11:**
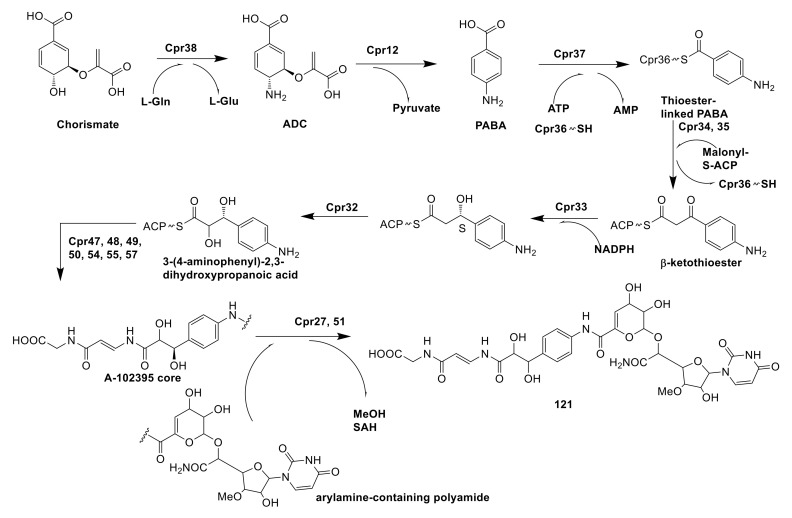
The biosynthetic pathway of A-102395 [84].

A-102395 (**121**) was a capuramycin-type nucleoside antibiotics possessed high specific chemical features, which were isolated from *Amycolatopsis* sp. SANK 60206. By synthase encoded by Cpr38, chorismate was catalyzed to form 4-amino-4-deoxychorismate (ADC), which subsequently catalyzed elimination of pyruvate by aminotransferase (Cpr12) to form para-aminobenzoic acid (PABA). Catalyzed by actinomycin synthetase (Cpr37), PABA became activated acyl-adenylate and combined with the free-standing carrier protein (Cpr36) to yield the thioester-linked PABA. Under the synergic catalyzation of ketosynthase (Cpr34) and chain length factor (Cpr35), the thioester-linked PABA as a recipient was decarboxylatively condensed with malonyl-S-acyl carrier protein (ACP) to form β-ketothioester. That β-ketothioester was reduced by 3-oxoacyl-ACP reductase Cpr33 and then hydroxylated by luciferase-like monooxygenase Cpr32 to form 3-(4-aminophenyl)-2,3-dihydroxypropanoic acid. The next step was polyamide biosynthesis, in which 3-(4-aminophenyl)-2,3-dihydroxypropanoic acid was catalyzed by a serious of enzymes including a hydrophilic amino acid (Cpr54), two carrier proteins (Cpr48 and 55), a condensation domain protein (Cpr47), and three transglutaminase-like proteins (Cpr49, 50 and 57) to form an A-102395 core. The coupling of the arylamine-containing polyamide to the A-102395 core was catalyzed by carboxyl methyltransferase (Cpr27) and MitI transacylase (Cpr51) [84]. However, the mechanism of Cpr51 has not been proven and needs further research (Figure 11).

**Figure 12:**
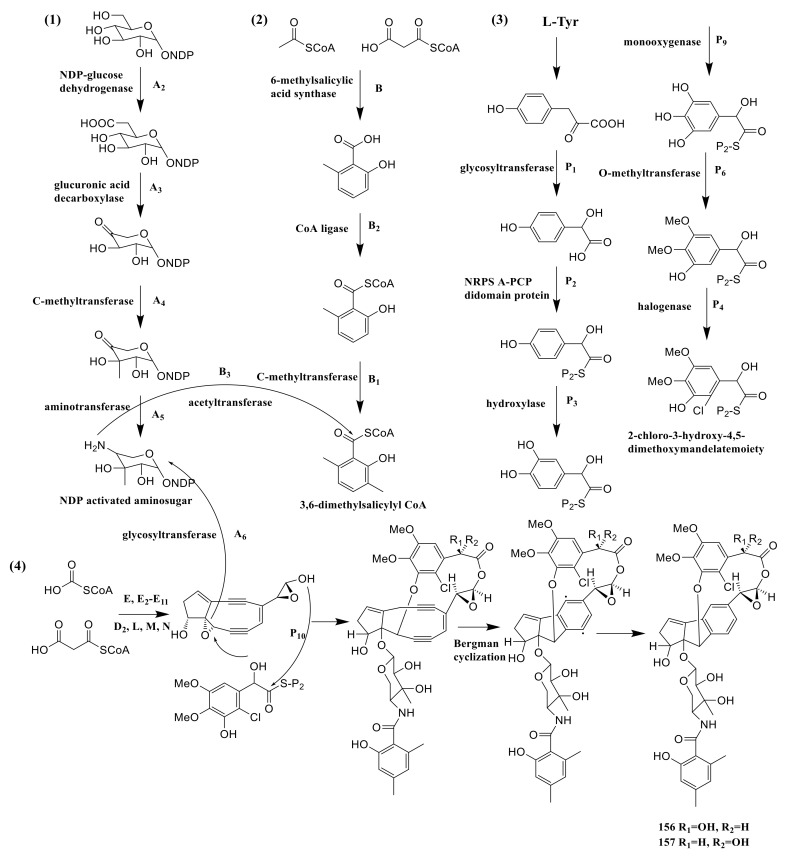
The biosynthetic pathway of amycolamycins A and B [55].

The strain *Amycolatopsis* sp. HCa4 possessed *acm* gene of amycolamycins A and B, which spanned a ∼76 kb contiguous DNA region. The Acm A_2_, A_3_, A_4_ and A_5_ provided NDP-glucose dehydrogenase, glucuronic acid decarboxylase, C-methyltransferase and aminotransferase. These enzymes catalyzed the NDP-glucose to NDP activated aminosugar. The 6-methylsalicylic acid synthase, CoA ligase and C-methyltransferase were encoded by Acm B, B_2_ and B_1_, which catalyzed three successive steps starting from acetyl-CoA and malonyl-CoA to 3,6- dimethylsalicylyl CoA. The genes of Acm P_1_, P_2_, P_3_, P_9_, P_6_ and P_4_ encoded the glycosyltransferase, NRPS A-PCP didomain protein, hydroxylase, monooxygenase, O-methyltransferase and halogenase. These enzymes catalyzed six successive steps converting p-hydroxyphenylpyruvate to 2-chloro-3-hydroxy-4,5-dimethoxymandelate moiety. Acetyl-CoA and malonyl-CoA were catalyzed to form enediyne core by a series of enzymes, which were encoded by E, E_2_-E_11_, D_2_, L, M and N. The next step needed B_3_ (acetyltransferase) to connect NDP activated aminosugar to 3,6-dimethylsalicylyl CoA. The NDP activated aminosugar and 2-chloro-3-hydroxy-4,5-dimethoxymandelate moiety were then connected to the enediyne core, which needs the Acm A_6_ (acetyltransferase) and Acm P_10_ (type II condensation enzyme), respectively. The connection product was transformed into compounds **156** and **157** by bergman cyclization (Figure 12) [55].

### 5.2. Chemical Synthesis, Semi-Synthesis and Biosynthesis of the Derivatives

#### 5.2.1. Chemical Synthesis of DHM2EQ

**Figure 13:**
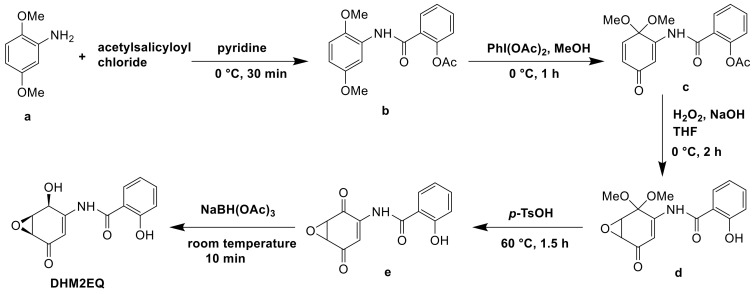
The chemical synthesis pathway of DHM2EQ [85].

DHM2EQ was a derivative of epoxyquinomicin C and possessed greater strong inhibitory activity on type II collagen-induced arthritis than epoxyquinomicin C. The derivative was synthesized from 2,5-dimethoxyaniline in 5 steps via chemical synthesis. In pyridine, 2,5-dimethoxyaniline (a) and acetylsalicyloyl chloride were coupled to give salicylamide (b). In methanol, compound (b) was oxidized into quinone monoketal (c) by iodobenzenediacetate. Under deprotection of the phenolic acetyl group, epoxidation of (c) in aqueous THF with alkaline hydrogen peroxide gave epoxide (d). Compound (d) was reduced by NaBH4 yield (e) and the deprotection of compound (e) with p-TsOH gave DHM2EQ (Figure 13) [85].

#### 5.2.2. Semi-Synthesis of 24-Desmethylrifampicin

**Figure 14:**
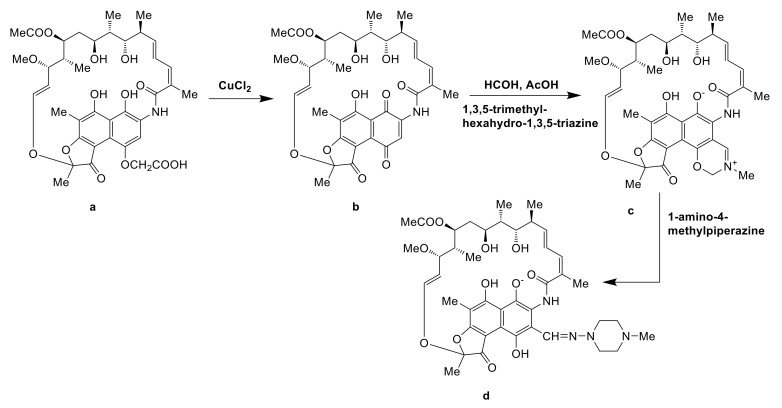
The semi-synthesis pathway of 24-desmethylrifampicin [86,87].

24-Desmethylrifampicin (d) was a semi-synthetic derivative of rifamycin B and 24-desmethylrifamycin B (a) was semi-synthetic precursor of (d). Nigam et al. replaced the acyltransferase domain of module 6 of rifamycin polyketide synthase (rifAT6) with that of module 2 of rapamycin polyketide synthase (rapAT2) to gain a mutant *A. mediterranei* S699 DCO#34, which could produce 24-desmethylrifamycin B (a). 24-Desmethylrifamycin S (b) was the oxidation product of compound (a) using CuCl_2_ as catalyst. Compound (b) was then treated with paraformaldehyde and 1,3,5-trimethyl-hexahydro-1,3,5-triazine in acetic acid to gain 3-methyl-1,3-oxazino(5,6-c)-24-desmethylrifamycin (c), which was subsequently treated with 1-amino-4-methylpiperazine to give 24-desmethylrifampicin (d) [86]. Nirjara et al. uncovered that the damage of RifP, RifQ, transport cascade was an essential reason of the low yield of only 20 mg/L of compound (a). They thought the production of compound (a) could be increased by blocking RifQ to restore the function of RifP in the future (Figure 14) [87].

#### 5.2.3. Biosynthesis of CDCHD

**Figure 15:**
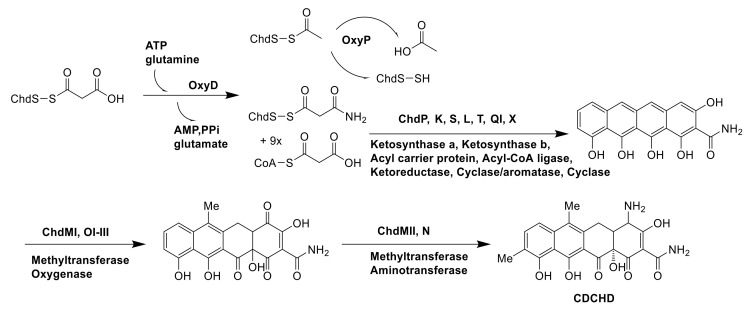
The biosynthesis pathway of CDCHD [88,89].

Chelocardin (CHD) isolated from *A. sulphurea* was a structurally atypical tetracycline [2]. CHD possessed excellent anti-microbial activity with little toxicity. 2-Carboxamido-2-deacetyl-chelocardin (CDCHD) was a derivative of CDH by introducing oxyD (amidotransferase) and oxyP (thiolase) genes from *Streptomyces rimosus* otc gene cluster into *A. sulphurea*. The production of CDCHD was very low when only introduced OxyD into *A. sulphurea* because OxyP could suppress priming of CDH by removing the competing acetyl units [88]. Then, the CDH gene cluster took over the rest of reaction [89]. These two genes worked together to change the main product from CHD to CDCHD (Figure 15).

## 6. Conclusions

This review summarizes the various chemical structures and biological activities of 159 compounds isolated from *Amycolatopsis* species inhabiting soil, insects, lichen, islands, the marine and plants between 1990–2020. A total of 45 compounds possessed bioactivities, of which 32 compounds have glycosides and 31 compounds have cyclic skeletons. Thus, the novel compounds with glycosides and cyclic skeletons should be considered by researchers. For example, compound **51**, the homolog of **49** and **50**, lacked glycoside and showed 5- to 100-fold less cytotoxicity [26]. The multitudinous secondary metabolites of the genus *Amycolatopsis* represent great research value and deserve further investigation. On the other hand, the genus of *Amycolatosis* could metabolize a variety of carbon sources and grow in a wide temperature range, which provides the possibility for them to become important biotechnological tools. It has been proven that this genus has great potential in degrading plastics, treating heavy metals, and biotransformation [68]. More researches are needed to transform these potentials into applications to solve practical problems to benefit mankind.

The study of biosynthetic pathway is a crucial process for excavating bioactive natural products. However, people are more willing to study the biosynthesis and mechanism of action of vancomycin, rifamycin and their derivatives. There are relatively fewer studies on the biosynthesis of other bioactive compounds and more attention is needed to be paid to researchers. In the course of the biosynthetic pathway study, a series of tools, for example, antiSMASH [90] or PRISM [91], have been fully exploited, which could derive a prediction of natural products, including the enzymes, regulatory genes and biosynthetic genes et al. through the genome sequencing results. We could also use these tools to reveal sufficiently more silent biosynthetic gene clusters and uncover more and more new interesting bioactive natural products. The biosynthetic potency of *Amycolatopsis* species is evidenced to be massive and this genus possesses many silent biosynthetic gene clusters waiting to be found [92]. Recently, Pan et al. obtained two new compounds, amycolapeptins A and B by combined-cultivating two strains of *Amycoaltopsis* sp. 26-4 and *Tsukamurella pulmonis* TP-B0596 for the first time, while they could not be discovered in a monoculture of *Amycoaltopsis* sp. 26-4 [93], which provided a new path for the cultivation of *Amycolatopsis*.

In conclusion, the research on the genus *Amycolatopsis* needs to be further considered in-depth. Most of all, the mechanism of action and biosynthetic regulatory genes of potent active compounds deserve to be deeply explored since they could determine the utility value of these compounds. Derivatives sometimes tend to have stronger activity so that more study might be focused on the structural modification of secondary metabolites for providing more analogues to be screened for antibiotics. In addition, compounds with excellent bioactivity that have been discovered should be solved for mass production due to their promising medicinal application. The potential ecological effects of *Amycolatopsis* species should be also taken seriously. The environmental pollution problem might be solved in some ways by thoroughly excavating the biofunction of the strains. In the future, we firmly believe that the genus *Amycolatopsis* will show its expansive utilization and serve for pharmaceutical area and environmental protection.

## Figures and Tables

**Table 1:** Secondary metabolites with sources, CAS registry numbers, and habitats from the genus *Amycolatopsis* during 1990–2020.

Structure Types	Compounds	Sources	CAS Registry Numbers	Habitats (T/M ^b^)	Refs.
Polyphenols	Kigamicin A (**1**)	*Amycolatopsis* sp. ML630-mF1	680571-49-7	Soil (T)	[13]
	Kigamicin B (**2**)	*Amycolatopsis* sp. ML630-mF1	680571-50-0	Soil (T)	[13]
	Kigamicin C (**3**)	*Amycolatopsis* sp. ML630-mF1	680571-51-1	Soil (T)	[13]
	Kigamicin D (**4**)	*Amycolatopsis* sp. ML630-mF1	680571-52-2	Soil (T)	[13]
	Kigamicin E (**5**)	*Amycolatopsis* sp. ML630-mF1	680571-53-3	Soil (T)	[13]
	Amexanthomycin A (**6**)	*A. mediterranei* S699 ∆rifA	/^a^	-^c^	[14]
	Amexanthomycin B (**7**)	*A.mediterranei* S699 ∆rifA	/	-	[14]
	Amexanthomycin C (**8**)	*A. mediterranei* S699 ∆rifA	/	-	[14]
	Amexanthomycin D (**9**)	*A. mediterranei* S699 ∆rifA	/	-	[14]
	Amexanthomycin E (**10**)	*A. mediterranei* S699 ∆rifA	/	-	[14]
	Amexanthomycin F (**11**)	*A. mediterranei* S699 ∆rifA	/	-	[14]
	Amexanthomycin G (**12**)	*A. mediterranei* S699 ∆rifA	/	-	[14]
	Amexanthomycin H (**13/**)	*A. mediterranei* S699 ∆rifA	/	-	[14]
	Amexanthomycin I (**14**)	*A. mediterranei* S699 ∆rifA	/	-	[14]
	Amexanthomycin J (**15**)	*A. mediterranei* S699 ∆rifA	/	-	[14]
	Mutactimycin E (**16**)	*Amycolatopsis* sp. 17128	1125635-23-5	Soil (T)	[15]
	Mutactimycin A (**17**)	*Amycolatopsis* sp. 17128	131749-16-1	Soil (T)	[15]
	Mutactimycin D (**18**)	*Amycolatopsis* sp. 17128	138689-82-4	Soil (T)	[15]
	1-Methoxy-3-methyl-8-hydroxy-anthraquinone (**19**)	*A. thermoflava* SFMA-103	67116-22-7	Soil (T)	[16,17]
	7-*O*-Methyl-5-*O*-α-L-rhamnopyranosylgenestein (**20**)	*Amycolatopsis* sp. YIM 130642	/	*Squamarina* sp. (T)	[18]
	7-*O*-α-D-Arabinofuranosyl daidzein (**21**)	*Amycolatopsis* sp. YIM 130642	602329-64-6	*Squamarina* sp. (T)	[18]
	Prunetin (**22**)	*Amycolatopsis* sp. YIM 130642	552-59-0	*Squamarina* sp. (T)	[18]
	Kakkatin (**23**)	*Amycolatopsis* sp. YIM 130642	57960-04-0	*Squamarina* sp. (T)	[18]
	Isoformononetin (**24**)	*Amycolatopsis* sp. YIM 130642	486-63-5	*Squamarina* sp. (T)	[18]
	Genistein (**25**)	*Amycolatopsis* sp. YIM 130642	446-72-0	*Squamarina* sp. (T)	[18]
	Formononetin (**26**)	*Amycolatopsis* sp. YIM 130642	485-72-3	*Squamarina* sp. (T)	[18]
	Sorbicillin (**27**)	*Amycolatopsis* sp. YIM 130687	79950-85-9	*P*. *borreri* (T)	[19]
	Pradimicin-IRD (**28**)	*Amycolatopsis* sp. IRD-009	2226037-84-7	Soil (T)	[20]
	(2*R*,3*R*)-2-Hydroxy-8-*O*-methyltetrangomycin (**29**)	*Amycolatopsis* sp. Hca1	1391860-71-1	*O*. *chinensis* (T)	[21]
	(2*R*,3*R*)-2-Hydroxy-5-O-methyltetrangomycin (**30**)	*Amycolatopsis* sp. Hca1	1391860-72-2	*O*. *chinensis* (T)	[21]
	Amycomycin A (**31**)	*Amycolatopsis* sp. Hca1	1415935-15-7	*O*. *chinensis* (T)	[22]
	Amycomycin B (**32**)	*Amycolatopsis* sp. Hca1	1415935-16-8	*O*. *chinensis* (T)	[22]
	Tetrangomycin (**33**)	*Amycolatopsis* sp. Hca1	7351-08-8	*O*. *chinensis* (T)	[21]
	Pd116779 (**34**)	*Amycolatopsis* sp. Hca1	102674-89-5	*O*. *chinensis* (T)	[21]
	Tetrangulol (**35**)	*Amycolatopsis* sp. Hca1	7414-92-8	*O*. *chinensis* (T)	[21]
	X-14881e (**36**)	*Amycolatopsis* sp. Hca1	85178-50-3	*O*. *chinensis* (T)	[21]
	Sakyomicin B (**37**)	*Amycolatopsis* sp. Hca1	86470-27-1	*O*. *chinensis* (T)	[21]
	Tetracyclinone (**38**)	*Amycolatopsis* sp. Hca1	86413-78-7	*O*. *chinensis* (T)	[21]
	Sakyomicin A (**39**)	*Amycolatopsis* sp. Hca1	86413-75-4	*O*. *chinensis* (T)	[21]
	Sakyomicin C (**40**)	*Amycolatopsis* sp. Hca1	86413-76-5	*O*. *chinensis* (T)	[21]
	Amycofuran (**41**)	*A. saalfeldensis*	/	Sponge (M)	[23]
Linear polyketides	ECO-0501 (**42**)	*A. orientalis* ATCC 43,491	848087-04-7	-	[24]
	Modified analogs of ECO-0501 (**43–47**)	*A. orientalis* ATCC 43,491	848087-07-0, 848087-06-9, 848087-08-1, 848087-09-2, 921224-72-8	-	[24]
	Vancoresmycin (**48**)	*Amycolatopsis* sp. ST 101170	268728-82-1	-	[25]
Macrolides	Amycolatopsin A (**49**)	*Amycolatopsis* sp. MST-108494	2209112-96-7	Soil (T)	[26]
	Amycolatopsin B (**50**)	*Amycolatopsis* sp. MST-108494	2209112-97-8	Soil (T)	[26]
	Amycolatopsin C (**51**)	*Amycolatopsis* sp. MST-108494	2209112-98-9	Soil (T)	[26]
	2′-*O*-Succinyl-apoptolidin A (**52**)	*Amycolatopsis* sp. ICBB 8242	1778681-11-0	Borneo (M)	[27]
	3′-*O*-Succinyl-apoptolidin A (**53**)	*Amycolatopsis* sp. ICBB 8242	1778681-12-1	Borneo (M)	[27]
	Apoptolidin A (**54**)	*Amycolatopsis* sp. ICBB 8242	194874-06-1	Borneo (M)	[27]
	Apoptolidin B (**55**)	*Amycolatopsis* sp. ICBB 8242	861994-72-1	Borneo (M)	[27]
	Apoptolidin C (**56**)	*Amycolatopsis* sp. ICBB 8242	861994-73-2	Borneo (M)	[27]
	Apoptolidin D (**57**)	*Amycolatopsis* sp. ICBB 8242	929641-83-8	Borneo (M)	[27]
	Isoapoptolidin A (**58**)	*Amycolatopsis* sp. ICBB 8242	476647-30-0	Borneo (M)	[27]
Macrolactams	Rifamycinoside A (**59**)	*A. mediterranei* S699	2329704-84-7	-	[28]
	Rifamycinoside B (**60**)	*A. mediterranei* S699	2329704-85-8	-	[28]
	28-Desmethyl-28-hydroxyrifamycin W (**61**)	*A. mediterranei* S699	2329704-86-9	-	[28]
	27,28-Epoxy-28-desmethylrifamycin W (**62**)	*A. mediterranei* S699	2329704-87-0	-	[28]
	30-Hydroxyrifamycin W hemiacetal (**63**)	*A. mediterranei* S699	2329704-88-1	-	[28]
	20-Hydroxyrifamycin S (**64**)	*A. mediterranei* S699	/	-	[28]
	Rifamycin S (**65**)	*A. mediterranei* S699	13553-79-2	-	[28]
	16,17-Dehydrorifamycin G (**66**)	*A. mediterranei* S699	75922-16-6	-	[28]
	Rifamycin O (**67**)	*A. mediterranei* S699	14487-05-9	-	[28]
	Rifamycin Z (**68**)	*A. mediterranei* S699	79486-49-0	-	[28]
	Rifamycin W (**69**)	*A. mediterranei* S699	53904-81-7	-	[28]
	Rifamorpholine A (**70**)	*Amycolatopsis* sp. HCa4	2101982-41-4	*L*. *migratoria* (T)	[29]
	Rifamorpholine B (**71**)	*Amycolatopsis* sp. HCa4	2101982-45-8	*L*. *migratoria* (T)	[29]
	Rifamorpholine C (**72**)	*Amycolatopsis* sp. HCa4	2101982-52-7	*L*. *migratoria* (T)	[29]
	Rifamorpholine D (**73**)	*Amycolatopsis* sp. HCa4	2101982-58-3	*L*. *migratoria* (T)	[29]
	Rifamorpholine E (**74**)	*Amycolatopsis* sp. HCa4	2101982-62-9	*L*. *migratoria* (T)	[29]
	Macrotermycin A (**75**)	*Amycolatopsis* sp. M39	1311284-73-7	*M*. *natalensis* (T)	[30]
	Macrotermycin B (**76**)	*Amycolatopsis* sp. M39	2095035-09-7	*M*. *natalensis* (T)	[30]
	Macrotermycin C (**77**)	*Amycolatopsis* sp. M39	2095035-10-0	*M*. *natalensis* (T)	[30]
	Macrotermycin D (**78**)	*Amycolatopsis* sp. M39	2095035-11-1	*M*. *natalensis* (T)	[30]
	Ansamycin (**79**)	*A. alba* DSM 44262	2256052-40-9	-	[31]
Thiazolyl peptides	Nocathiacin I (**80**)	*A. fastidiosa*	214044-52-7	-	[32,33,34]
	Nocathiacin II (**81**)	*A. fastidiosa*	256230-46-3	-	[32,33]
	Nocathiacin III (**82**)	*A. fastidiosa*	256230-47-4	-	[32,33]
	Nocathiacin IV (**83**)	*A. fastidiosa*	400650-12-6	-	[32,33]
	Thiazomycin (**84**)	*A. fastidiosa*	905946-70-5	-	[32,33,34]
	Thiazomycin A (**85**)	*A. fastidiosa*	905978-04-3	-	[33]
	Mj347-81f4 b (**86**)	*A. fastidiosa*	214044-53-8	-	[35]
	Thiazomycin B (**87**)	*A. fastidiosa*	905946-73-8	-	[35]
	Thiazomycin C (**88**)	*A. fastidiosa*	851664-21-6	-	[35]
	Thiazomycin D (**89**)	*A. fastidiosa*	905946-71-6	-	[35]
	Thiazomycin E_1_ (**90**)	*A. fastidiosa*	905946-72-7	-	[35]
	Thiazomycin E_2_ (**91**)	*A. fastidiosa*	905946-75-0	-	[35]
	Thiazomycin E_3_ (**92**)	*A. fastidiosa*	905946-76-1	-	[35]
	Thioamycolamide A (**93**)	*Amycolatopsis* sp. 26−4	/	Iriomote Island (T)	[36]
	Thioamycolamide B (**94**)	*Amycolatopsis* sp. 26−4	/	Iriomote Island (T)	[36]
	Thioamycolamide C (**95**)	*Amycolatopsis* sp. 26−4	/	Iriomote Island (T)	[36]
	Thioamycolamide D (**96**)	*Amycolatopsis* sp. 26−4	/	Iriomote Island (T)	[36]
	Thioamycolamide E (**97**)	*Amycolatopsis* sp. 26−4	/	Iriomote Island (T)	[36]
Cyclic peptides	PRG-A (**98**)	*Amycolatopsis* sp. ML1-hF4	421547-03-7	Soil (T)	[37]
	PRG-B (**99**)	*Amycolatopsis* sp. ML1-hF4	2112795-88-5	Soil (T)	[38]
	PRG-C (**100**)	*Amycolatopsis* sp. ML1-hF4	2112795-89-6	Soil (T)	[38]
	PRG-D (**101**)	*Amycolatopsis* sp. ML1-hF4	2112795-90-9	Soil (T)	[38]
	Valgamicin A (**102**)	*Amycolatopsis* sp. ML1-hF4	2271221-78-2	Soil (T)	[39]
	Valgamicin C (**103**)	*Amycolatopsis* sp. ML1-hF4	2271221-79-3	Soil (T)	[39]
	Valgamicin T (**104**)	*Amycolatopsis* sp. ML1-hF4	2271221-80-6	Soil (T)	[39]
	Valgamicin V (**105**)	*Amycolatopsis* sp. ML1-hF4	2271221-81-7	Soil (T)	[39]
Glycopeptides	Chloroorienticin A (**106**)	*A. orientalis* PA-45052	118395-73-6	-	[40]
	Chloroorienticin B (**107**)	*A. orientalis* PA-45052	118373-81-2	-	[40]
	Chloroorienticin C (**108**)	*A. orientalis* PA-45052	118373-82-3	-	[40]
	Chloroorienticin D (**109**)	*A. orientalis* PA-45052	118373-83-4	-	[40]
	Chloroorienticin E (**110**)	*A. orientalis* PA-45052	118373-84-5	-	[40]
	Orienticin A (**111**)	*A. orientalis* PA-45052	111073-20-2	-	[40]
	Orienticin D (**112**)	*A. orientalis* PA-45052	112848-46-1	-	[40]
	Vancomycin (**113**)	*A. orientalis* PA-45052	1404-90-6	-	[40]
	Vancomycin aglycone (**114**)	*A. orientalis* PA-45052	82198-76-3	-	[40]
	MM 47,761 (**115**)	*A. orientalis* NCBI 12608	126985-51-1	-	[41]
	MM 49,721 (**116**)	*A. orientalis* NCBI 12608	126985-52-2	-	[41]
	Eremomycin B (**117**)	*A. orientalis* subsp. *Eremomycini*	1193347-07-7	-	[42]
	Eremomycin (**118**)	*A. orientalis* subsp. *Eremomycini*	110865-90-2	-	[42]
Amide derivatives	Albachelin (**119**)	*A. alba*	2055362-14-4	-	[43]
	Albisporachelin (**120**)	*A. albispora* WP1^T^	/	Sediment (M)	[44]
	A-102395 (**121**)	*Amycolatopsis* sp. SANK 60206	1003904-77-5	Soil (T)	[45]
	Amycocyclopiazonic acid (**122**)	*A. saalfeldensis*	/	Sponge (M)	[23]
	Amycolactam (**123**)	*A. saalfeldensis*	/	Sponge (M)	[23]
	Carbamothioic S-acid (**124**)	*A. alba* DSM 44262*∆abm*9	/	-	[46]
	Amycophthalazinone A (**125**)	*Amycolatopsis* sp. YIM 130642	/	*Squamarina* sp. (T)	[18]
	2-Pyruvoylaminobenzamide (**126**)	*Amycolatopsis* sp. YIM 130687	18326-62-0	*P*. *borreri* (T)	[19]
	(−)-Chrysogine (**127**)	*Amycolatopsis* sp. YIM 130687	42599-89-3	*P*. *borreri* (T)	[19]
	4-(3-Methylbut-2-enyloxy) benzamide (**128**)	*Amycolatopsis* sp. YIM 130687	116208-80-1	*P*. *borreri* (T)	[19]
	Acetotryptamide (**129**)	*Amycolatopsis* sp. YIM 130687	1016-47-3	*P*. *borreri* (T)	[19]
	2-Acetamidophenol (**130**)	*Amycolatopsis* sp. YIM 130687	614-80-2	*P*. *borreri* (T)	[19]
	Anthranilic acid (**131**)	*Amycolatopsis* sp. YIM 130687	118-92-3	*P*. *borreri* (T)	[19]
	Phenacetamide (**132**)	*Amycolatopsis* sp. YIM 130687	103-81-1	*P*. *borreri* (T)	[19]
	2-Carbamoyl-3-hydroxy-1,4-naphthoquinone (**133**)	*Amycolatopsis* sp. YIM 130687	103646-20-4	*P*. *borreri* (T)	[19]
	Echinosporin (**134**)	*Amycolatopsis* sp. YIM PH20520	79127-35-8	Soil (T)	[47]
	7-Deoxyechinosporin (**135**)	*Amycolatopsis* sp. YIM PH20520	431945-10-7	Soil (T)	[47]
	Dipyrimicin A (**136**)	*Amycolatopsis* sp. K16-0194	1235020-43-5	-	[48]
	Dipyrimicin B (**137**)	*Amycolatopsis* sp. K16-0194	1332747-97-3	-	[48]
	1-(10-Aminodecyl) pyridinium (**138**)	*A. alba* var. nov. DVR D4	1421439-67-9	Sediment (M)	[49]
	Siderochelin A (**139**)	*Amycolatopsis* sp. LZ149	77550-87-9	Cynodon dactylon (T)	[50]
	Siderochelin B (**140**)	*Amycolatopsis* sp. LZ149	2252179-56-7	Cynodon dactylon (T)	[50]
	Siderochelin C (**141**)	*Amycolatopsis* sp. LZ149	2252179-55-6	Cynodon dactylon (T)	[50]
	Siderochelin D (**142**)	*Amycolatopsis* sp. LZ149	2249835-41-2	Cynodon dactylon (T)	[50]
	Epoxyquinomicin A (**143**)	*A. sulphurea* MK299-95F4	175448-31-4	Soil (T)	[51]
	Epoxyquinomicin B (**144**)	*A. sulphurea* MK299-95F4	175448-32-5	Soil (T)	[51]
	Epoxyquinomicin C (**145**)	*A. sulphurea* MK299-95F4	200496-85-1	Soil (T)	[51]
	Epoxyquinomicin D (**146**)	*A. sulphurea* MK299-95F4	200496-86-2	Soil (T)	[51]
Glycoside derivatives	Tigloside (**147**)	*Amycolatopsis* sp. NN0 21702	216590-44-2	-	[52]
	2,2′-Di-*O*-β-D-glucopyranosyl-α-D- glucopyranosyl α-D- glucopyranoside (**148**)	*Amycolatopsis* sp. NN0 21702	/	-	[52]
	Actinotetraose I (**149**)	*Amycolatopsis* sp. HCa1	1427319-31-0	*O*. *chinensis* (T)	[53]
	Actinotetraose J (**150**)	*Amycolatopsis* sp. HCa1	1427319-40-1	*O*. *chinensis* (T)	[53]
	Actinotetraose K (**151**)	*Amycolatopsis* sp. HCa1	1427319-41-2	*O*. *chinensis* (T)	[53]
	Actinotetraose A (**152**)	*Amycolatopsis* sp. HCa1	1421368-85-5	*O*. *chinensis* (T)	[53]
	Actinotetraose B (**153**)	*Amycolatopsis* sp. HCa1	1421368-86-6	*O*. *chinensis* (T)	[53]
	Actinotetraose C (**154**)	*Amycolatopsis* sp. HCa1	1421368-87-7	*O*. *chinensis* (T)	[53]
	Actinotetraose L (**155**)	*Amycolatopsis* sp. HCa1	216590-44-2	*O*. *chinensis* (T)	[54]
Enediyne derivatives	Amycolamycin A (**156**)	*Amycolatopsis* sp. HCa4	2243041-65-6	*L*. *migratoria* (T)	[55]
	Amycolamycin B (**157**)	*Amycolatopsis* sp. HCa4	2243041-66-7	*L*. *migratoria* (T)	[55]
Sesquiterpenes	(*E*)-3-methyl-5-(2,6,6-trimthyl-3-oxocyclohex-1-enyl) pent-2-enoic acid (**158**)	*A. alba* DSM 44262	2247139-21-3	-	[31]
	(*E*)-3-methyl-5-(2,6,6- trimthyl-4-oxocyclohex-2-enyl) pent-2-en-oic acid (**159**)	*A. alba* DSM 44262	2256051-20-2	-	[31]

^a^ The CAS registry number was not found; ^b^ T: terrestrial environment; M: marine environment; ^c^ The habitat was not mentioned.

**Table 2:** The antimicrobial, cytotoxic and other bioactivities of secondary metabolites from *Amycolatopsis*.

Activity Types	Compounds	Bioactivities (MIC, μg/mL or IC_50_, μM)	Refs.
Antimicrobial activities	Kigamicins A–E (**1–5**)	MRSA (0.03–0.22 μM)	[13]
	Mutactimycin E (**16**)	MRSA, *S. pneumonia*, *E. faecium* (1–16 μg/mL)	[15]
	7-*O*-Methyl-5-*O*-α-L-rhamnopyranosylgenestein (**20**) and 7-*O*-α-D-arabinofuranosyl daidzein (21)	*C. albicans*, *E. coli*, MRSA, *S. aureus*, and *S. typhi* (32–256 μg/mL)	[18]
	Pradimicin-IRD (**28**)	*S. agalactiae*, *S. aureus* and *P. aeruginosa* (3.15 μg/mL)	[20]
	ECO-0501 (**42**)	MRSA (0.125–0.25 μg/mL)	[24]
	Vancoresmycin (**48**)	MRSA, *E. faecium*, *E. faecalis* (0.05 μM)	[25]
	Amycolatopsins A, C (**49**, **51**)	*M. bovis* (0.4 and 2.7 μM) *M. tuberculosis* (4.4 and 5.7 μM)	[26]
	Rifamorpholine B (**71**)	MRSA, *S. aureus*, *S. pyogenes*, *B. subtilis*, **M. luteus** (0.5–4.0 μM)	[29]
	Rifamorpholine D (**73**)	MRSA, *S. aureus*, *S. pyogenes*, *B. subtilis*, **M. luteus** (1.0–8.0 μM)	[29]
	Macrotermycin A (**75**)	*B. subtilis*, *S. aureus*, *S. cerevisiae*, *C. albicans* (1.0–10 μg/mL)	[29]
	Macrotermycin C (**77**)	*B. subtilis*, *S. aureus*, *S. cerevisiae*, *C. albicans* (10–25 μg/mL)	[29]
	Thiazomycin (**84**) and thiazomycins A–D (**85**, **87**–**89**)	*S. aureus*, *E. faecalis*, *S. pneumonia* and their drug-resistant type (0.002–0.06 μg/mL)	[31,32,33,34]
	PRG-A, C (**98**, **100**)	MRSA, *E. faecalis*, **M. luteus**, *B. subtilis* (0.72 μM)	[37,38]
	PRG-B, D (**99**, **101**)	MRSA, *E. faecalis*, **M. luteus**, *B. subtilis* (5.62–23.37 μM)	[38]
	Chloroorienticins A–E (**106–110**)	*S. aureus* JC-1 and MRSA (0.2–0.78 μg/mL)	[40]
	Vancomycin (**113**)	*S. aureus* JC-1 (0.78 μg/mL) and MRSA (1.58 μg/mL)	[40]
	MM 47,761 (**115**) and MM 49,721 (**116**)	*B. subtilis* ATCC6633, *C. xerosis* NCTC9755, **M. luteus** NCTC8340, *S. aureus*, *S. saprophyticus* FL1, *S. epidermidis* 60137, *S. pyogenes* CN10, *S. agalactiae* Hester, *S. sanguis* ATCC 10556, *S. faecalis* I (0.5–8 μg/mL)	[41]
	Amycophthalazinone A (**125**)	*S. aureus*, *S. typhi*, *C. albicans* (6.92–13.84 μM)	[18]
	Echinosporin (**134**)	*F. oxysporum*, *F. solani*, *A. panax,* and *P. herbarum* (32–128 μg/mL)	[47]
	7-deoxyechinosporin (**135**)	*F. oxysporum*, *F. solani*, *A. panax,* and *P. herbarum* (32–128 μg/mL)	[47]
	Dipyrimicin A (**136**)	*S. cerevisiae*, *Kocuria rhizophila*, *B. subtilis*, *Escherichia coli* NIHJ, *Xanthomonas campestris pv. oryzae* KB 88 (16–21 mm)	[48]
	Siderochelin A (**139**)	*Bacillus pumilus*, *B. subtilis*, *E. coli* and *S. aureus* (10–15 mm)	[50]
	Epoxyquinomicins A (**143**) and B (**144**)	*M. luteus* IFO3333, *M. luteus* PCI1001 (3.12–6.25 μg/mL)	[51]
Cytotoxic activity	Kigamicin D (**4**)	Mouse tumor cell lines LB32T, L-1210, EL-4, P388D1, B16BL6, FS3, Colon26 (0.95 μM)	[13]
	1-methoxy-3-methyl-8-hydroxy-anthraquinone (**19**)	Lung cancer (10.3 µM) Lymphoblastic leukemia cells (16.98 µM)	[17]
	Pradimicin-IRD (**28**)	HCT-116 (0.8 μM), MM 200 (2.7 μM), MCF-7 (1.55 μM), RPE (1.48 μM)	[20]
	Tetrangomycin (**33**)	HeLa cells (0.27 μM)	[21]
	Pd116779 (**34**)	HeLa cells (0.11 μM)	[21]
	Sakyomicin A (**39**)	HeLa cells (0.56 μM)	[21]
	Sakyomicin C (**40**)	HeLa cells (0.39 μM)	[21]
	Amycolatopsins A, B (**49**, **50**)	SW620 (0.08 and 0.14 μM) NCIH-460 (1.2 and 0.28 μM)	[26]
	3′-O-succinyl-apoptolidin A (**52**)	H292 cells (0.09 μM)	[27]
	2′-O-succinyl-apoptolidin A (**53**)	H292 cells (0.08 μM)	[27]
	Apoptolidin A (**54**)	H292 cells (0.02 μM), HeLa cells (0.04 μM)	[27]
	Thioamycolamides A, D (**93, 96**)	HT1080(11.94 and 21.22 μM) HeLa S3(6.53 and 9.34 μM)	[27]
	Valgamicin V (**105**)	MIA Paca 2, HGC-27, GSS, 5637, NCI-H1650, NB16, ME-180, HSC-490 (6.6–21.6 μM)	[39]
	Amycolactam (**123**)	SNU638 (0.8 μM) HCT116 (2.0 μM)	[23]
	Dipyrimicin A (**136**)	Hela 3S, HT29, A549, H1299, Panc1, THP-1, Jarkat, HL-60 (3.9–9.4 μM)	[48]
	Dipyrimicin B (**137**)	H1299 cell (6.8 ± 3.3 μM)	[48]
	Amycolamycin A (**156**)	M231 (7.9 μM)	[55]
Other activities	Amexanthomycins A–C (**6–8**)	Inhibiting human DNA Topo IIα	[14]
	1-methoxy-3-methyl-8-hydroxy-anthraquinone (**19**)	Antioxidant Anti-hyperglycemic	[17]
	Rifamycinoside A and B (**59–60**), 28-Desmethyl-28-hydroxyrifamycin W (**61**),30-Hydroxyrifamycin W hemiacetal (**63**), Rifamycin O (**67**)	Inhibiting human DNA Topo I (50 and 100 μM)	[28]
	Rifamycinoside A and B (**59–60**), 28-Desmethyl-28-hydroxyrifamycin W (**61**),30-Hydroxyrifamycin W hemiacetal (**63**), Rifamycin S, O and Z (**65**, **67** and **68**)	Inhibiting human DNA Topo IIα (50 μM)	[28]
	20-hydroxyrifamycin S (**64**)	Inducing G2/M phase arrest Causing DNA damage in HCT116	[28]
	A-102395 (**121**)	Inhibiting bacterial translocase I (0.01 μM)	[45]
	Epoxyquinomicins C (**145**) and D (**146**)	Inhibiting type II collagen-induced arthritis	[51]

## Data Availability

All data in this article is openly available without any restrictions.
